# Is Raman the best strategy towards the development of non-invasive continuous glucose monitoring devices for diabetes management?

**DOI:** 10.3389/fchem.2022.994272

**Published:** 2022-09-26

**Authors:** Biagio Todaro, Filippo Begarani, Federica Sartori, Stefano Luin

**Affiliations:** ^1^ NEST Laboratory, Scuola Normale Superiore Pisa, Italy; ^2^ P.B.L. SRL, Solignano, PR, Italy; ^3^ Omnidermal Biomedics SRL, Solignano, PR, Italy; ^4^ NEST, Istituto Nanoscienze, CNR, Pisa, Italy

**Keywords:** diabetes, Raman spectroscopy, spectral data processing, glycaemia monitoring devices, wearable continuous non-invasive sensors, blood glucose

## Abstract

Diabetes has no well-established cure; thus, its management is critical for avoiding severe health complications involving multiple organs. This requires frequent glycaemia monitoring, and the gold standards for this are fingerstick tests. During the last decades, several blood-withdrawal-free platforms have been being studied to replace this test and to improve significantly the quality of life of people with diabetes (PWD). Devices estimating glycaemia level targeting blood or biofluids such as tears, saliva, breath and sweat, are gaining attention; however, most are not reliable, user-friendly and/or cheap. Given the complexity of the topic and the rise of diabetes, a careful analysis is essential to track scientific and industrial progresses in developing diabetes management systems. Here, we summarize the emerging blood glucose level (BGL) measurement methods and report some examples of devices which have been under development in the last decades, discussing the reasons for them not reaching the market or not being really non-invasive and continuous. After discussing more in depth the history of Raman spectroscopy-based researches and devices for BGL measurements, we will examine if this technique could have the potential for the development of a user-friendly, miniaturized, non-invasive and continuous blood glucose-monitoring device, which can operate reliably, without inter-patient variability, over sustained periods.

## 1 Background

Diabetes is a lifelong disease that affects more than 400 millions of people worldwide (WHO. Diabetes, 2022). Emerging reports from the International Diabetes Federation state that diabetes is set to rise very fast, estimating 700 millions of cases in the next 25 years (IDF Diabetes Atlas, 2019). Among the various types of diabetes, all characterized by high blood glucose levels, the main two types are type 1 diabetes, an autoimmune condition where the pancreas produces little or no insulin, and type 2 diabetes, a metabolic disorder that results in hyperglycaemia due to insulin resistance. Diabetes, and related risk factors such as microvascular (retinopathy, nephropathy, and neuropathy) and macrovascular metabolic disorders, is so widespread that it has been defined “the epidemic of the century” (Kharroubi, 2015). According to the European parameters (Blood Sugar Level Ranges, 2019), the plasma blood glucose level (BGL) of a healthy person under fasting conditions fluctuates during the day between approximately 99.0 and 124.2 mg/dl (5.5–6.9 mmol/L) with maximum levels of up to 140.4 mg/dl (7.8 mmol/L) 2 h post-prandial. Conversely, the plasma BGL of a diabetic person under fasting conditions fluctuates during the day between approximately 124.2 and 198.2 mg/dl (6.9–11.0 mmol/L) with maximum levels up to 250.0 mg/dl (13.7 mmol/L) 2 h post-prandial. In this contest, an adequate therapeutic treatment has a pivotal role to avoid life-threatening health complications, such as risks of heart disease, kidney failure, blindness, up to hypoglycaemic or hyperglycaemic coma. While subcutaneous injection of insulin or oral/nasal administration of sugar-lowering drugs have been proved as the crucial drug delivery systems over the last years, recent pharmacotherapeutic approaches, exploiting i.e., nanotechnologies, provide nowadays important alternatives into resolving some of the most several limitations of conventional anti-diabetes medications (Veiseh et al., 2015; Todaro et al., 2022).

However, despite these technological improvements, it remains difficult to maintain long-lasting ideal blood glucose levels, and people with diabetes (PWD) must monitor the BGL several times per day.

In this regards, great progresses have been made during the past 60 years regarding BGL sensors. Weller and co-workers pioneered the first continuous *in vivo* BGL sensing in 1960 (Weller et al., 1960). Starting from that year, several finger-stick device have been developed, from the traditional Accu-Chek (Roche Diagnostics GmbH, Germany), OneTouch (LifeScan Inc., United States), Freestyle Optium Neo (Abbott Diabetes Care Inc., United States), Contour Next, and Contour Next One (Ascensia Diabetes Care Inc., Canada) to the most novel technologies, such as a device based on a wireless smart pen that can automatically calculate required insulin (Huang et al., 2022). The conception of the traditional finger-stick for blood testing method has further represented a cornerstone of the research efforts, even if nowadays this tool is considered obsolete because painful and time consuming, sometimes resulting in poor compliance and bearing the risk of infections. In the quest for painless alternatives, researchers are attempting with extensive efforts to develop a fully Non-Invasive Continuous Blood Glucose Level (NICBGL) sensing device since the Eighties. (John L. Smith, 2021). Several technologies (Heineman and Jensen, 2006) have been and are being developed globally towards this end; until now, however, a completely user-friendly, cheap, small and reliable NICBGL device is lacking.

This review is organized as follows. Section 2 provides an overview of current optical/wave-based/remote measurement methods for BGL estimation; the main advantages and drawbacks of the technologies are discussed. Section 3 presents a summary of the Raman spectroscopy advances over the years. The main Raman features, advantages, disadvantages as well as the emerging results in Raman biosensors are examined. In Section 4 we discuss more deeply the requirements for the ideal BGL measurement device and how the various techniques can approach this result, provide an intuitive explanation for the data analysis methods, highlight the aspects that are important in particular for avoiding (too frequent) recalibrations, and discuss the advantages of Raman over the other possible techniques, presenting therefore also the perspectives of this work. The manuscript ends with a short conclusions section.

## 2 Blood glucose monitoring devices

### 2.1 Overview

The history of blood glucose monitoring (BGM) devices began in the mid-twentieth-century when Leland C. Clark Jr., considered the “father of biosensors,” designed the first-generation enzymatic glucose biosensor made to monitor glucose during cardiac surgery. Due to its higher specificity, accuracy and reliability in comparison to the previous chemical/enzymatic tests (i.e., Trommer’s test, 1841; Fehling’s test, 1848; or Benedict’s test, 1908), this device was one of the major breakthroughs in glucose measurement (Dziergowska et al., 2019). Thereafter, many other (invasive and non-continuous) enzyme-based biosensors as well as other continuous glucose monitoring (CGM) devices have been or are being developed exploiting several techniques for measuring various glucose physical parameters (Harman-Boehm et al., 2009; Oliver et al., 2009; Clarke and Foster, 2012; Villena Gonzales et al., 2019; Bolla and Priefer, 2020).

**FIGURE 1:**
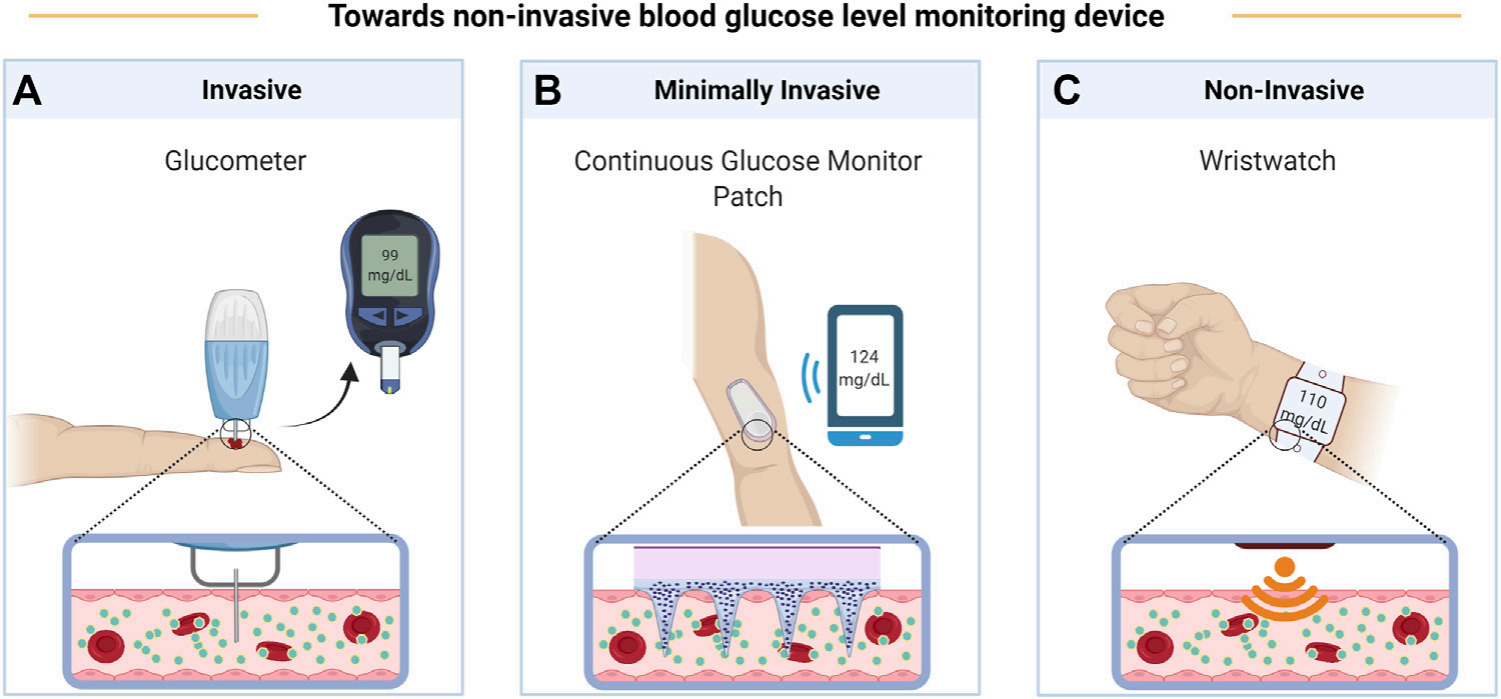
Evolution of BGL monitoring devices from invasive glucometer **(A)**, to minimally invasive continuous glucose monitoring patch **(B)**, and finally to non-invasive wristwatch **(C)**. Created with BioRender.com.

**FIGURE 2:**
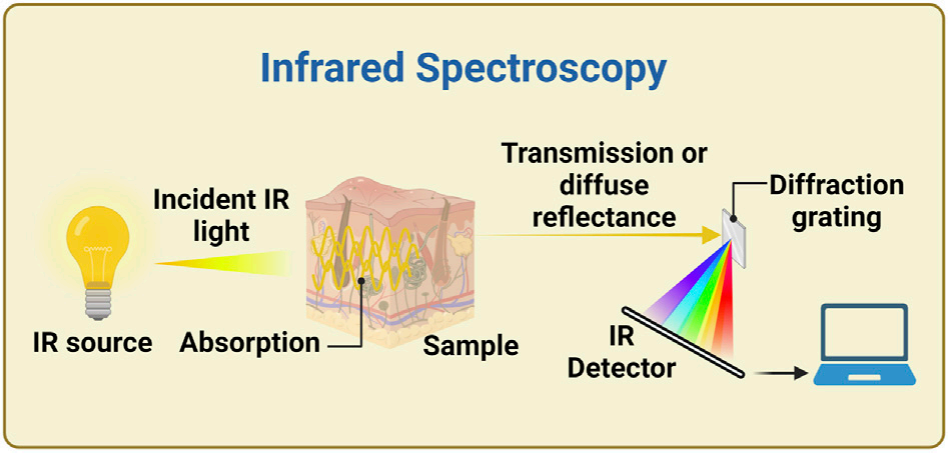
Example of experimental setup for infrared spectroscopy. A continuous IR source generates light over a wide range of infrared wavelengths, which irradiates the sample. The light is collected in transmission or diffuse reflectance mode, and the spectrum is measured thanks to a diffraction grating, in this example. Alternative configurations use a Fourier transform interferometer (FTIR) instead of the dispersive element, or use a monochromator or other methods for selecting the wavelength bands between IR source and sample. Created with BioRender.com.

**FIGURE 3:**
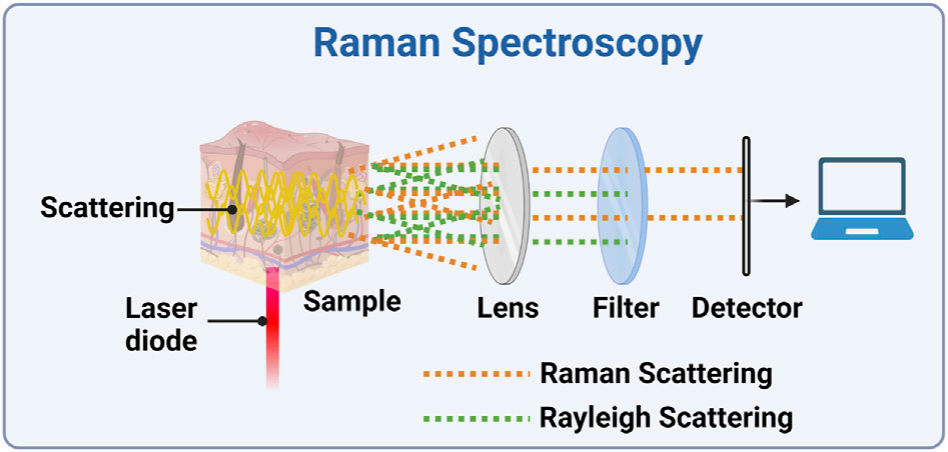
Experimental setup for a Raman spectrometer. A laser diode is focused onto the sample and the scattered light is collected by a lens (alternatively, the backscattered light could be collected). The Rayleigh scattered light is then and blocked, while the Raman scattered light can be detected and its spectrum can be analysed. Created with BioRender.com.

Measuring the concentration of an analyte requires detecting a signal which is related to its amount, and knowing or determining the functional relationship. The signal is usually generated through the interaction of a field or of other molecules (the “source”) with some physical-chemical characteristic of the analyte. The source may interact with the analyte in the body because:

1) a quantity of the analyte proportional to its concentration is extracted (Figure 1A),
2) something is inserted inside the tissue of interest (Figure 1B),
3) source and signal can pass through the tissues (Figure 1C).

The different possible sources and signals, linked with the measured physico-chemical characteristic of the analyte, characterize the different possible methods and techniques. In the case of the BGM devices reviewed here, extraction of fluids (case 1) is exploited in finger-pricking methods, in Reverse Iontophoresis (RI, Section 2.7) and Sonophoresis Technology (ST, 2.8) with subsequent enzymatic or surface enhanced Raman scattering (SERS) quantification of glucose (2.6 or 2.3.1, respectively); insertion of a minimally invasive device (case 2) can be used in Enzymatic Detection Technology (EDT, 2.6), in Fluorescence Technology (FT, 2.9), or again with SERS (2.3.1).

Case 3) is maybe the most promising for completely non-invasive CGM devices, and sources and signals are usually pressure/movement waves (“sound”) and/or electromagnetic fields (“light”). The various techniques based on electromagnetic fields differ for the frequency at which these vary, and for how the signal is detected. At the lowest frequencies, we will discuss Bioimpedance Spectroscopy (BS, Section 2.11) and ElectroMagnetic Sensing (EMS, 2.14).

Increasing the frequency, there are the different optical spectroscopy techniques, based on absorption by, emission from, or scattering by the analyte. These three different measures would require different geometries, but the possibly relatively low penetration of the used electromagnetic radiation in tissues, and the requirement for a CGM device to be small and wearable, most often constrain the geometry to be (almost) in back-scattering, i.e. with the signal detected very close to the source. In particular, absorption is usually measured as “diffuse reflectance” (or remission), i.e., exploiting the random scattering of penetrating radiation from tissues. Absorption can be observed also by considering the effect of the energy absorbed by the molecules, i.e. the local heating which can be measured directly (sometimes called photothermal spectroscopy) or by the generated pressure wave (photoacoustic spectroscopy, see Section 2.5). Depending on the measured regions of the glucose absorption spectrum, the related devices are based on Near, Mid, and Far Infrared Spectroscopy (NIRS, MIRS, and FIRS, see Section 2.2), or Millimetre and Microwave Sensing (MMS, 2.4). Regarding light scattering: coherent diffused radiation is measured in Optical Coherence Tomography (OCT, Section 2.12), where glucose concentration can be determined because of its influence on the refractive index of biofluids; on the other hand, a vibrational spectrum of glucose can be measured by looking at the inelastic light scattering in Raman Spectroscopy (RS, Sections 2.3, 3). Finally, there are devices that exploit more than one technique and/or measure also quantities different from glycaemia (e.g., to correct its obtained values); these are MultiTechnologies (MT) devices (Section 2.13), among which the ones measuring Metabolic Heat Conformation (MHC), based on various spectroscopies and possibly on gravitometry (for detecting movements, e.g., caused by heart pulse).

In the following, we will provide examples of BGM devices that are available on the market or are or have recently been under development, and we will explain the main complications of each technique. A more comprehensive research on BGM devices on the market or currently under development is presented in (Shang et al., 2022).

### 2.2 Infrared spectroscopy

In general, absorption of infrared radiation usually causes a variation in molecular vibrational states. The vibrational spectrum of a molecule is usually composed by relatively sharp peaks, with energies precisely depending on the structure of the molecule (spectral fingerprint region); therefore, studying infrared absorption spectra allows drawing information on distinctive features of the molecules. Infrared spectroscopy can be used as a quantitative analytical method, since the absorption is proportional to the intensity of the incident light and to the concentration of the considered molecule. This, together with the high selectivity of the method, allows the quantitative determination of an analyte in a complex mixture with limited or no prior separation.

A basic configuration for an IR measurement is shown in Figure 2. A Fourier transform infrared interferometer (FTIR) is often used as the dispersive element especially in the mid and far infrared, because it is more efficient than gratings (and prisms, where there could also be high absorption), especially in producing a higher signal to noise ratio (SNR) (Tozzini and Luin, 2012). However, measurements with this instrument are usually slower, measures on only some subsets of wavelengths are not feasible, and the moving part makes them difficult to implement in a wearable device.

#### 2.2.1 Near infrared spectroscopy

NIR absorption spectroscopy is an analysis technique based on the interactions between matter and electromagnetic radiation with wavelengths between ∼700 and ∼2,500 nm, corresponding to energies between ∼14,000 and ∼4,000 cm^−1^. In this region there is the so-called NIR optical window, i.e. a range of wavelengths (∼650–1,350 nm) at which light penetrates tissues the most, thanks to the low absorption of water and haemoglobin.

In the NIR region, the active modes are actually overtone or combination bands, with somehow broader spectra. Moreover, there are some peaks in the absorption spectrum of water, also within the optical window; increasing the concentration of an analyte will decrease the absorption of water in these regions, causing negative peaks in the differential absorption between the studied solution and water (water volume-displacement effects). Moreover, other changes in the absorption spectra could arise because of differences in water hydrogen bonds, due to interactions with the solute.

Often, NIR is combined with photoplethysmography (PPG). PPG is a non-invasive optical technique introduced for the first time in 1937 by Hertzman, who was the first suggesting that a pulse oximeter pleth (Plethysmograph), an instrument for measuring blood or air fluctuations, may be used to detect blood volume changes in the skin’s vessels. A typical PPG device is composed by a LED, which emits infrared light on the skin, and by a photodetector, which captures and measures the diffused-reflected light. The resultant PPG tracks were intensely studied for several decades until 2015, when for the first time the PPG signals were analysed by means of hard spectral data processing methods for the first BGL estimation. (Monte-Moreno, 2011; Ramasahayam et al., 2015).

Advantages of the NIR technique are that it is suitable in the presence of interfering substances such as plastic, glass and water, needs relatively low-cost materials and the required photoconductive detectors are highly sensitive. Only three NIR-based devices are currently available on the market, i.e. the Combo Glucometer (Cnoga Medical, Israel), where NIR is combined PPG, one from the Tech4Life entreprise (United States) and the HELO Extense (World Global Network, United States); these are little devices providing a non-invasive glucose detection through the finger. Unfortunately, they need frequent and personalized calibration, thus monitoring the glucose in a non-continuous manner, and often do not measure directly glucose spectral characteristics. (Pfützner et al., 2018; Segman, 2018; Vahlsing et al., 2018; Villena Gonzales et al., 2019).

WizmiTM (Wear2b Ltd., Israel), LifeLeaf^®^ (LifePlus, United States) and two devices from the Polytechnic University of Catalunya (Spain) and the Karunya University (India) are under development (Monte-Moreno, 2011; Hadar et al., 2019; Villena Gonzales et al., 2019; Shang et al., 2022; MEET LIFELEAF, 2022 ^®^). The first one could offer a non-invasive and continuous glucose monitoring when applied to the arm wrist and the other three could harness the PPG technique when applied on the wrist, finger and forearm, respectively.

Other devices, such as NBM-200G (OrSense Ltd., Israel) and Diasensor 1,000 (Biocontrol Technology, United States), or GluControl^®^ GC300 (Samsung Fine Chemicals Co., Ltd. & Arith. Med Gmb. H, United States) and one from TouchTrack Pro, were withdrawn or never released, respectively, because of their weak sensitivity and stability, mostly caused by the high scattering of tissues. Indeed, almost all NIR devices suffer from the need to perform frequent recalibrations and from poor selectivity (Oliver et al., 2009; Villena Gonzales et al., 2019; Bolla and Priefer, 2020). Even if there are spectra peaks whose intensity is directly proportional to the concentration of the analyte (glucose), its concentration is very low and there can be many reasons for changes in NIR spectra (e.g., the effects on water discussed above). Accordingly, complex machine learning model and multivariate calibration models, such as partial least squares (PLS) regression, support vector regression or Monte-Carlo simulation, are required for extracting a quantification of glucose in the presence of other physiological substances and tissue components (water, haemoglobin, proteins, fat, etc.) (Kino et al., 2016; Althobaiti and Al-Naib, 2021). For better understanding the state of the art of the non-invasively measured NIR signals from tissue, see studies from the Heise group, who reviewed the progress in emerging glucose monitoring techniques exploiting photoplethysmography within the visible and near-infrared range (Delbeck et al., 2019; Heise et al., 2021).

#### 2.2.2 Mid infrared spectroscopy

Mid Infrared Spectroscopy (MIRS) allows collecting spectra where the contribution of different moieties (including blood glucose) are clearer if compared to NIRS, and therefore it can be more specific (Oliver et al., 2009). MIRS is a vibrational spectroscopy technique that exploits radiation in the mid-infrared region (2.5–25 μm, corresponding to 4,000–400 cm^−1^), where there is lower scattering from the tissues, and high absorption in the so-called fingerprint region of organic molecules. Conversely, the main problem of this technology is related to the fact that the light has limited penetration in tissues (100 μm approximately) for the strong water absorption, thus making necessary the use of expensive complementary technologies. No devices are currently available, but some products are under development. The start-up DiaMonTech AG (Germany) is developing D-Band, D-Pocket and D-Base, three analogous devices exploiting “photothermal detection” of MIR absorption spectra upon excitation with a tuneable mid-infrared quantum cascade laser (QCL) (Shang et al., 2022; DiaMonTech, 2022; DiaMonTech: Non-Invasive Blood Glucose Monitoring.); these products are based on the researches of Mäntele group, who stated that photoacoustic and photothermal detection seem to provide high accuracy in following glucose absorbance signal, overcoming cross-sensitivities and interpersonal variation of skin glucose level measurements (Lubinski et al., 2021). Indeed, also a device under development by the Swiss Federal Institute of Technology (Switzerland) exploits powerful but expensive QCL MIR sources and photoacoustic detection. (Kottmann et al., 2016; Villena Gonzales et al., 2019). Another device (under development in Tohoku University, Japan) is a minimally invasive device applicable on the inner lips, harnessing an attenuated total internal multireflection geometry (Kino et al., 2016; Villena Gonzales et al., 2019). However, the use of attenuated total reflection for this kind of measures has been criticized due to penetration depth limitations (Delbeck and Heise, 2021).

#### 2.2.3 Far infrared spectroscopy

At still higher wavelengths there is far infrared radiation (FIR), having wavelengths of ∼25–1,000 μm (sub-millimetre waves), corresponding to energies of ∼400–10 cm^−1^ or to frequencies between ∼12 and 0.3 THz, and therefore it is also known as terahertz radiation. FIR spectroscopy (FIRS), for instance, is less sensitive towards scattering compared to NIR and MIR and does not require frequent calibration. However, the scattered radiation intensity depends on skin temperature and skin thickness, and strong water absorption makes extremely difficult to identify other analytes, such as glucose, in the sample. Therefore, no instrument based on this technology is known to exist or be under development.

Terahertz Time-Domain Spectroscopy (THz-TDS), and Time of Flight (TOF), are nevertheless promising emerging technologies. Despite the long measurement time and the low spatial and depth resolution, these technologies seems more suitable than the IR spectroscopy for the identification of glucose, whatever in solid-state or in aqueous solutions. (Song et al., 2018). Furthermore, the possibility to study broad frequency ranges with a single ultrashort pulse and to make complex permittivity measurement with a single scan are two added values of this approach, which could be exploited in the near future in BGL assessment (Villena Gonzales et al., 2019).

### 2.3 Raman spectroscopy

Raman spectroscopy allows exploring the vibrational transitions addressed also by MIRS and down to part of the FIRS range; like those, it is a non-destructive optical technique useful for obtaining analytical information on the (bio)chemical composition of a sample (Pandey et al., 2017). This is irradiated by a monochromatic light source usually in the visible or NIR range (typically a laser beam), and the photons scattered by the sample are recorded (Figure 3) (Oliver et al., 2009; Dziergowska et al., 2019). There are two types of scattered light, namely Rayleigh (elastic scattering, at the same frequency of the incident light), and the weaker Raman scattering (anelastic scattering, with different frequencies than the incident light) (Wróbel, 2016; Villena Gonzales et al., 2019). The energy difference between the two scattering modes is the Raman shift, which corresponds to the energy of an excitation of the considered system. As already stated, these excitations are usually vibrational modes, and their spectrum form a unique “fingerprint” of the specific chemical substance (Pandey et al., 2017). Usually, the Stokes Raman peaks (at lower energies than the one of the source photons) are measured, since the Anti-Stokes signal (at higher energies) is much weaker at room and physiological temperatures. Atkins and co-workers (2017) reviewed the literature in the field, listing the published Raman spectroscopy studies of haemoglobin and red blood cells, white blood cells, platelets, plasma and serum, and whole blood (Atkins et al., 2017). RS is so far one of the most promising technologies due to its numerous advantages. The main one is the fact that, by choosing the source in the red region of the spectrum or in the NIR, both source and signal can be within the optical window for tissue transparency, and this allows a good penetration depth (up to millimetres) into human tissue. In this configuration, also an FTIR can be used for obtaining the spectra with the same advantages and disadvantages discussed in Section 2.2; it has been used with 1,064 nm laser excitation also for minimizing the disturbance of fluorescence background (Wang Q. et al., 2021). Other advantages are its unequivocal detection capability (and subsequently high specificity to glucose) without issues of photostability, minimal interference by temperature changes and water presence, and the high amount of developed methods for quantitative data analysis. As a result, Raman spectroscopy has significant potentials to provide precious data in several clinical assessment processes, such as diagnosing cancer, infections or neurodegenerative diseases, as well as for non-invasive BGM (Parlatan et al., 2019).

A more complete review of the research on RS for measuring BGL is reported in Section 3, and the information reported here are for better comparison with the other techniques. Considering devices, apart C8 Medisensors (C8 Medisensors inc., United States), which was never released nominally due to the absence of capital to finalize the design, GlucoBeam (RSP System A/S, Denmark) seems to be the only BGM Raman device under development (Lundsgaard-Nielsen et al., 2018; Villena Gonzales et al., 2019). The authors claim that clinical tests on 600 participants have been already performed and some intrinsic limiting factors typical of Raman, such as the usually low SNR as well as the interference of signals caused by the high complexity of biological tissues and by a strong fluorescence background, have been resolved. However, unstable laser wavelength and intensity, long collection time and accuracy issues need to be solved before future clinical applications (Li et al., 2019; Kang et al., 2020; Pleus et al., 2021; Wróbel et al., 2021).

#### 2.3.1 Surface enhanced Raman spectroscopy

Another strategy to overcome the weakness of Raman signals exploits the high enhancement of the signal when molecules are adsorbed, or at least very close, to metallic surfaces with nanometric features (Tozzini and Luin, 2012). This is exploited in surface enhanced Raman scattering (SERS), where there is both an enhancement of the electromagnetic field for its “storage” in nanoplasmonic modes, and a “chemical” enhancement, due to the interactions of the molecular orbitals with the electronic states in the metal. This technique provides both excellent detection sensitivity (down to single-molecule detection) and the high selectivity of RS (SERS fingerprint spectrum). Reports proved that SERS-active plasmonic devices could represent a useful platform for molecular recognition/sensing inside the body. For instance, Park et al. developed a plasmonic microneedle array coated with gold nanorods and with the pH-sensitive molecule 4-mercaptobenzoic acid as a platform for pH sensing in *ex vivo* human skin (Park et al., 2019). A wearable sensing platform, formed by a flexible SERS-active plasmonic silver superlattice metasurface as key sensing component and by a flexible electronic system for iontophoresis (see Section 2.7), was able to automatically extract sweat and analytes from the body and to reveal trace-amounts of drugs and glucose inside the body (Wang Y. et al., 2021). Over the last years, SERS strategies have opened novel horizons also in diabetes management, as the development of 1) silver-coated intracutaneous microneedle to detect glucose concentrations ranging from 5 to 150 mM (Yuen and Liu, 2014), 2) gold nanorods SERS probe embedded in two-component self-assembled monolayers consisting of 3-mercaptophenylboronic acid and 1-decanethiol, to measure the glucose concentration in the range 2–16 mM (Torul et al., 2014), 3) a subcutaneous glucose sensor by tracking the SERS emission of mercaptophenylboronic acid (MPBA), (Li et al., 2015), 4) composite of gold nanoparticles (AuNPs) onto two-dimensional (2D) nanosheet metalloporphyrinic metal−organic framework (MOF), used for the detection of glucose in saliva (Hu et al., 2020), 5) a glucose sensor based on a poly(methyl methacrylate) (PMMA) microneedle array coated with silver nanoparticles (Ag NPs) to achieve mice intradermal measurements (Ju et al., 2020). Although SERS has been extensively studied for glycaemia management and the usage of such sensors will probably expand more in the coming years, no device is on the market yet. Because of the requirement for the SERS-active surface to touch the analyte, SERS can be used in external or extracted fluids or, in the last reported examples, is not a completely noninvasive technology. Indeed, subcutaneous injection of metal materials can produce toxicity, appropriate microneedles are costly and can easily cause permanent skin damages, and in any case *in-vivo* efficacy has been not completely demonstrated. (Asharani et al., 2008; Ma et al., 2011; Moore et al., 2018; Wang et al., 2022).

### 2.4 Millimetre and microwave sensing

Millimetre and Microwave sensing (MMS) can be based on reflection, transmission, resonance perturbation and/or radar techniques using electromagnetic fields oscillating at ∼30–300 GHz and ∼3–30 GHz respectively; in any case, the glucose concentration could be inferred by the dependence of the permittivity of blood and tissues on the glucose concentration, which causes different interactions with the electromagnetic waves (Saha et al., 2017; Shaker et al., 2018). This radiation can penetrate deeper in tissues than more energetic ones, allowing to reach areas with more circulating blood and more glucose, and enhancing therefore the sensitivity for this molecule; always for the deep penetration of these waves, it is possible to perform glucose measurements on different areas of body (hand, abdomen, ear lobe and other portions of skin). Several universities and companies from Europe and United States (MediWise, University of Waterloo and Google, University of Cardiff, Caltech University and University of Erlangen-Nuremberg) are currently developing devices for a non-invasive glycemia tracking exploiting the previously mentioned advantages (Choi et al., 2017; Shaker et al., 2018; Villena Gonzales et al., 2019; Omer et al., 2020). However, it is worth noting that these devices are not suitable for continuous glucose monitoring (i.e. Glucowise™ or Google Soli, both equipped with 60 GHz mm-wave radar, could be not user-friendly after a long exposure) and the penetration of signal can be affected from physiological parameters such as sweating, breathing and cardiac activity; thus, the glucose concentration measurement could be inaccurate.

### 2.5 PhotoAcoustic spectroscopy

**FIGURE 4:**
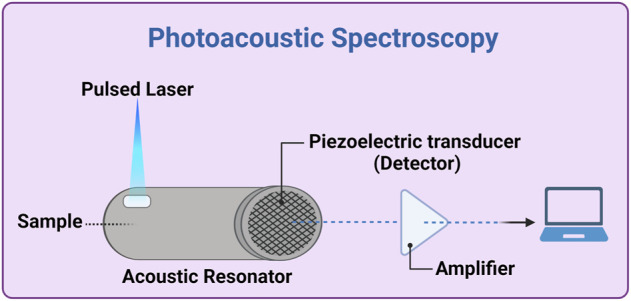
Photoacoustic spectroscopy experimental setup. A thermal expansion of the sample (inside or in contact with the acoustic resonator) is generated by a pulsed laser source (e.g., a quantum cascade -QC- laser). The generated acoustic waves propagate through the acoustic resonator and, after amplification and possibly ADC (analogic-digital converter), is analyzed by a computer. Created with BioRender.com.

This technology allows developing a relatively simple and compact sensor, employing a nanosecond-pulsed QCL or a modulated laser, with wavelength from the ultraviolet to the MIR ranges, and exploiting the fact that absorption of radiation by an analyte produces microscopically localized heating. This causes a fast and adiabatic thermal expansion of the sample and the generation of detectable acoustic waves as a consequence (Villena Gonzales et al., 2019). Therefore, absorbance spectra in any of the regions discussed above can be measured by detection with ultrasound detectors, and the variations of blood glucose level can be calculated similarly to the all-optical methods, with the advantage that the tissues mostly transmit sound waves (Figure 4). However, the signal may not be intense enough (low SNR) and it may be susceptible to temperature, motion, pulsation and surrounding acoustics; moreover, the integration time could be long (several minutes) (Oliver et al., 2009). Some examples of PAS-based devices under development have been reported above, especially in Section 2.2.2. Other possible examples are Aprise (Glucon, United States) and another device from the Electronics and Telecomm. Research Inst. of Korea (Republic of Korea), but they are almost unknown and not on the market (Kottmann et al., 2016; Sim et al., 2018; Dziergowska et al., 2019; Villena Gonzales et al., 2019).

### 2.6 Enzymatic detection technology

Enzymatic detection represents the first developed method to reveal glucose level. Through technologies exploiting enzymatic reactions, it is possible to provide a direct and efficient measurement not only from blood, but also from other biological fluids (e.g., tears and interstitial fluid) (Park et al., 2018). The commercially available CGM sensors based on enzymatic detection, without the typical long-term stability issue of enzyme, are: 1) Guardian Sensor 3 (Medtronic Plc., United States) (Cappon et al., 2017; Christiansen et al., 2017; Lee et al., 2021; Shang et al., 2022), 2) Dexcom G6 (DexCom, Inc., United States) (Cappon et al., 2017; Boscari et al., 2021, 2022; Lee et al., 2021; Shang et al., 2022), and 3) Free Style Libre (Abbott Ltd., United States) (Cappon et al., 2017; Blum, 2018; Galindo et al., 2020; Jafri et al., 2020; Lee et al., 2021; Shang et al., 2022). All three electrochemical sensors works by means of an enzymatic sensor, equipped with sterile and fluffy microneedles, which are subcutaneously inserted (on abdomen or upper arm), and the following automatic data sharing on smartphone. However, every 7, 10 and 14 days, respectively for Medtronic, Dexcom and Abbott, the sensors need to be replaced. In the last years, research institutes have been moving towards the development of similar but longer-life CGM devices. It is this case for: 1) K’Watch, a skin patch under development by PKvitality (France); 2) the devices under development by KTH Royal Inst. of Technology (Sweden), to be applied on the forearm; 3) mouthguard glucose sensor, from Tokyo Medical and Dental University (Japan) and 4) the biosensor platform from the iQ Group Global Ltd. (formerly iQnovate, Australia), exploiting the Organic Thin Film Transistor technology, both developed to monitor salivary glucose that is reported to be correlated with the BGL; 5) the device from Ulsan National Inst. of Science and Technology (South Korea) and 6) NovioSense from Novio Tech Campus (Netherlands), both exploiting eye glucose monitoring technology (Dastoor and Belcher, 2017; Kownacka et al., 2018; Park et al., 2018; Ribet et al., 2018; Villena Gonzales et al., 2019; Arakawa et al., 2020; Shang et al., 2022). Data on most of these devices are not in the public domain. However, it should be mentioned that, although all of them are able to check the glycaemia level with a painless scan instead of fingersticks, they are all considered minimally invasive devices (Ventrelli et al., 2015).

### 2.7 Reverse iontophoresis

Reverse Iontophoresis (RI) is a minimally invasive technique, based on an electrochemical apparatus applicable on the skin that can extract glucose from the interstitial fluid (ISF). Upon application of electrodes and then of an electric field, the flux of the target compound is possible thanks to passive diffusion and electroosmosis, which is a net movement of water across the skin from anode to cathode and results when the electric field is applied across the negatively-charged skin (the isoelectric point of human skin is around 4–4.5); glucose is then revealed through enzymatic methods (Dziergowska et al., 2019). The first two example of RI continue glucose monitoring devices were developed in the United Kingdom. SugarBEAT (Nemaura Medical, United Kingdom), available on the market, is a daily upper arm disposable sensor whilst another device from the University of Bath (United Kingdom), under development, seems to use a graphene-based transdermal platform. (Lipani et al., 2018; Villena Gonzales et al., 2019; Shang et al., 2022). The main advantages of devices employing a RI technology are that electrodes are not difficult to manufacture and they are easily applied to skin; in addition, there is a good correlation between glucose level in the ISF and in the blood under stable conditions. GluCall (K.M.H Co., Ltd., South Korea) is another RI device currently present on the market but not widely used due to its high degree of invasiveness. Common disadvantages of RI devices are high susceptibility to sweating, slowness in responding to rapid changes of glucose concentration, besides provoking skin irritation due to the passage of current. For these reasons, and for the low reliability, another RI continuous monitoring glucose device, named GlucoWatch (Cygnus Inc., United States) was withdrawn (Oliver et al., 2009; Villena Gonzales et al., 2019).

### 2.8 Sonophoresis technology

Sonophoresis is a well-known drug delivery method relying on low-frequency pressure waves to move molecules into and across the skin. Sonophoresis instruments induce a series of compression and expansion movements and enhance the permeability of the skin; by slight changes in the geometry, they can be used to extract ISF with glucose, similarly to RI, allowing a CGM by means of enzymatic methods (Oliver et al., 2009; Dziergowska et al., 2019). On the other side, determining the relationship between the extracted glucose quantity and the BGL is usually difficult, and can be susceptible to temperature and pressure variations, to environmental parameters, and to the presence of other compounds. Based on sonophoresis approach, the American company Echo Therapeutics is currently trying to develop “Symphony”, a CGM device catalogued as a minimally invasive technology, even if it seems to harness a user-friendly approach as there is no side-effect to skin (Villena Gonzales et al., 2019).

### 2.9 Fluorescence technology

Although an old patent based on an assumptive fluorescence of glucose excited at 308 nm is reported, glucose actually has no detectable fluorescence when excited in the visible or near-UV range (Khalil, 2004; Pullano et al., 2022). Fluorescence technologies make instead use of fluorophores that, once bound to the analyte (e.g., glucose), are able to emit fluorescent light with distinctive optical properties (mostly, excitation and detection wavelengths). Thus, the analyte concentration can be measured in terms of fluorescence intensity and/or decay times. Fluorescence technology is receiving much attention for its high sensitivity and specificity also in scattering media such as blood, skin layers or tears; however, it requires contact between the fluid to be analysed and the fluorophores, likewise enzymatic detection (Dziergowska et al., 2019). In this contest, Eversense^®^ CGM under-skin biosensor (Senseonics, United States) is today available on the market. Furthermore, another under-skin patch (from Profusa, inc., United States) and a contact lens, that can monitor the glucose concentration in tears, are currently under development (Chen et al., 2017; Badugu et al., 2018; Villena Gonzales et al., 2019; Jafri et al., 2020). The last one is based on an optical fluorescent sensor that monitor the BGL chemically through a boronic-acid derivative support containing the fluorophore; the higher the glucose amount, the more boronic acid converts from neutral to anionic form, and the bigger the spectral variations retrieved by the detector (Badugu et al., 2004, 2018; Gamsey et al., 2006). Despite their ability to reveal glucose at very low concentration (lower than 25 μM for Eversense^®^), none of these devices is currently widely used worldwide. As a matter of fact, they are not exempt from invasiveness: they need an “exogenous” fluorescence-based sensor/indicator in contact with the analyte, the foreign body containing all this is inserted within biological media, and this may lead to local tissue trauma and potential toxicity issues. In addition, they suffer from the intrinsic FT issues related to autofluorescence and its limitations associated with photostability/photobleaching. Because of these obstacles, the research has quickly moved towards less invasive CGM devices (Oliver et al., 2009; Villena Gonzales et al., 2019). Such is the case of DermalAbyss (Massachusetts Institute of Technology, United States), relying on a colour-based indicator for glucose concentration that can be used in a pioneering tissue-integrating tattoo (Vega et al., 2017). This is now under development and represents a promising biosensor for continuous monitoring, despite issues related to the very high sensitivity to local pH changes and/or oxygen levels.

### 2.10 Metabolic heat conformation

**FIGURE 5:**
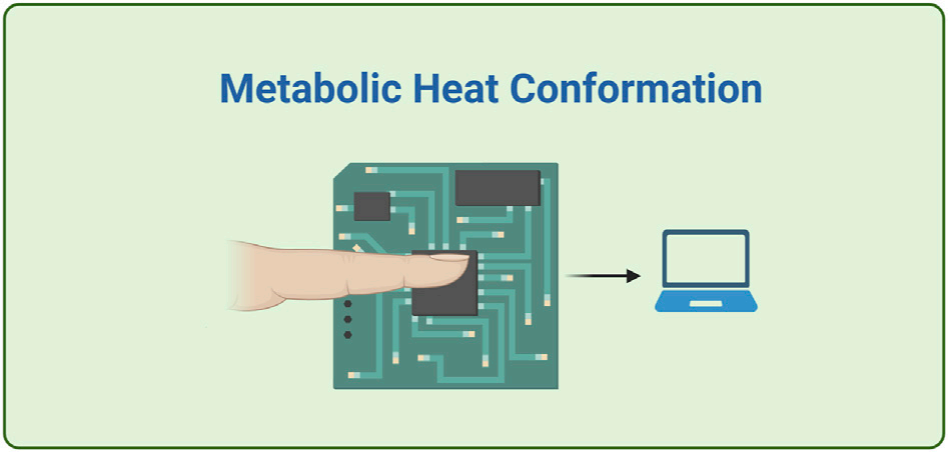
A schematics of a simplified MHC device, where the detection system transforms information about temperature, humidity, blood flow rate and degree of blood oxygen saturation into estimation of glucose level. Created with BioRender.com.

MHC technology consists in deducing glycaemia level from measurements of physiological indicators related to metabolic heat generation and local oxygen amount, such as pulse rate, oxyhaemoglobin saturation, heat metabolic rate and the blood flow volume, via well-known multi-wavelength (MIR/NIR range) spectroscopy methods (Figure 5) (Dziergowska et al., 2019; Villena Gonzales et al., 2019). GlucoGenius (ESER Health Care Digital Technology Co. Ltd., Taiwan) is the only available device on the market, while other non-invasive devices, one from Health-Care Computer (Japan) and G2 Mobile (Eser Digital, India), are under development. Hitachi Ltd. (Japan) announced in 2004 the development of a unique non-invasive blood sugar monitoring device for diabetics but it was never released (Okura et al., 2018; Villena Gonzales et al., 2019). The main issue of these tools is that they don’t provide a direct glucose monitoring; besides, they suffer of high sensitivity towards temperature and sweat; thus, MHC doesn’t seems a suitable technology for continuous glucose monitoring purpose (Bolla and Priefer, 2020).

### 2.11 Bioimpedance spectroscopy

Bioimpedance spectroscopy (BS) was a very popular technology in the early 2000s. It is supposedly based on the fact that the conductivity of the red blood cells membranes can be correlated with variation of blood glycaemia level, since the glucose concentration influences the sodium-potassium currents in cells (Oliver et al., 2009; Dziergowska et al., 2019). Pendra (Pendragon Medical Ltd., Switzerland) and Glucoband (Calisto Medical, Inc., United States) were the first two developed BS devices that allowed an easy CGM measurement on arm wrist cheaply (Villena Gonzales et al., 2019). On the other side, the conductivity was measured by means of the application of a current on the skin for a long time, which could cause irritation. Moreover, as MHC technology, BS is sensitive to variations of temperature, to motion, to sweat and to water content. For these reasons, Pendra and Glucoband were withdrawn few years after their release and are considered obsolete (Huang et al., 2020).

### 2.12 Optical coherence tomography

Optical Coherence Tomography (OCT) is a medical imaging technique, initially born as an ocular diagnostic test and evolved later as tomographic imaging of different tissues also sensible to various analytes, such as glucose. OCT is typically a non-invasive method, characterized by a good SNR due to NIR light, deeply penetrating in the skin. Although it can suffer from tissue inhomogeneity, this technology does not seem susceptible to blood pressure, haematocrit and cardiac activity (Dziergowska et al., 2019; Villena Gonzales et al., 2019). There’s currently only one device from the National Cheng Kung University (Taiwan), considered however not suitable for continuous glucose monitoring (Chen et al., 2018; Villena Gonzales et al., 2019). Researchers are currently trying to overcome some OCT related issues, such as the high sensitivity to temperature changes of the skin and to motion, and the lack of chemical specificity for glucose (Oliver et al., 2009; Wróbel, 2016).

### 2.13 MultiTechnologies

The technologies already described are analytical methods used for the determination of glucose by using one or more of its intrinsic molecular properties, or by assessing the effects of glucose on the physical properties of blood and/or tissues. However, glucose determination with different methods presents different drawbacks and it is limited by the influence of interfering factors (which could be, e.g., temperature, humidity, motion of the sensor, or other physio-pathological parameters of the PWD). Accordingly, the development of a tool that combines several technologies may decrease the errors derived from each method separately, or can be used to determine the interfering factors in order to correct the glucose measure. Starting from 2009, researchers from the Integrity Applications (Israel) placed on the market GlucoTrack, apparently a combination of ultrasound, thermal and electromagnetic sensing device, a non-invasive glucose monitoring device intended for people with type 2 diabetes for use in home and home-alike environments. (Harman-Boehm et al., 2009; Villena Gonzales et al., 2019; Shang et al., 2022). For measuring BGL upon a personal calibration valid for 1 month, a personal ear clip, equipped with both sensors of hypo- and hyperglycaemia and calibration electronics, is attached to the earlobe. This latter is an interesting site since its accessibility and the abundant supply of blood to it. The device is lightweight, compact and safe and seems to achieve very high accuracy and precision in clinical trials due to combination of multiple technologies (Mohamad Yunos and Nordin, 2020). More recently, other technologies have been developed. Evia Medical Technologies Limited (Saudi Arabia), exploiting similar technology than GlucoTrack, has developed Egm1000™, an ear-lobe glucose monitoring device available for type 2 diabetes (Mosli and Madani, 2021). To date, GlucoTrack and Egm1000™ are not suitable for a CGM because they are not really wearable.

In another kind of multitechnology approach, the United States researchers Xu and Berry are currently trying to exploit graphene’s Raman spectroscopy (GRS) as a tool for reading the activity of a glucose oxidase (GOx) enzymatic-based detector or the response to glucose in an affinity-based detector; RS can indeed measure the changes in graphene dopant level and/or Fermi energy caused by these kinds of detectors without employing conventional electrical measurements (Ahmadianyazdi et al., 2022). The reported measurements have been done on fluids containing glucose; however, once device sensitivity, measurement uncertainty, sample-to-sample variation, and compatibility with biological fluids will be overcome, this glucose sensor could represent a complementary tool for the study of molecular interaction phenomena at the interface with graphene and a promising glycaemia continuous monitoring biosensor.

### 2.14 Other technologies

Besides the above described approaches, there are several other technologies that could be employed in BGL estimation but no BGM devices are currently on the market due to drawbacks mainly related to reliability, size or cost. Optical Polarimetry Technology (OPT) is an optical measurement method that relies on rotation of light polarization due to the optical activity of an analyte. The aqueous humour of the eye has been identified as the fittest tissue for BGL measurements with this technique, because of low protein and erythrocytes contents and therefore negligible scattering. However, OPT is not a front-line technology for glycaemia sensing purpose, firstly due to the intrinsic low optical activity of glucose. Furthermore, several challenges have yet to be addressed such as high sensitivity to interferences (other optically active compounds), temperature, pH changes and motion and the need of an external laser source and proper alignment with eye (Oliver et al., 2009; Dziergowska et al., 2019; Villena Gonzales et al., 2019; Bolla and Priefer, 2020).

Surface Plasmon Resonance Technology (SPRT) is a refractive index-based detection technology not suitable for BGL assessment since glucose and other low molecular weight compounds have insufficient mass to affect a measurable change in the refractive index. Moreover, this technique is very sensitive to temperature, sweat and motion, and the instruments are generally bulky and require a long calibration time (Villena Gonzales et al., 2019).

Electromagnetic sensing (EMS) is a low-cost, easily miniaturized and safe technology, which supposedly exploits the dependence on glucose of the magnetic permittivity of tissues. However, several challenges, such as the low glucose selectivity as well as the high sensitivity to temperature, have to be addressed before making EMS technology available for glycaemia measurement (Villena Gonzales et al., 2019; Bolla and Priefer, 2020).

Ultrasound Technology (UT) is a well-established technology, not harmful to cells, and highly sensitive for qualitative/quantitative analysis due to the long penetration through skin and other tissues of ultrasound waves. Its sensitivity to glucose is linked to the dependence of sound velocity on glucose concentration. However, UT is not a suitable approach for BGL measurement, due to the limited accuracy (pressure changes and temperature fluctuations can cause interferences, and therefore it is often coupled with NIRS) as well as the high cost (Villena Gonzales et al., 2019; Bolla and Priefer, 2020).

Occlusion Spectroscopy (OS), based on red light or NIR scattering often in the reflected-diffusion configuration, is suitable for the non-invasive measurement of arterial glucose. However, due to the susceptibility to many endovascular variables, such as pharmacological treatment, internal erythrocyte aggregation, free fatty acid concentration and chylomicrons (ULDLs), this technology cannot be used to develop BGM devices without a complex algorithm, still lacking, to extract glucose information (Shokrekhodaei and Quinones, 2020).

RadioFrequency Sensor Technology (RFST) is another approach to monitor the different levels of blood glucose concentration (Moore, 2009; Yilmaz et al., 2019; Yunos et al., 2021). Compared to sensors with different mechanisms, Alertgy^®^ (Alertgy Ltd., United States) and UBAND™ (Know Labs, United States) RF devices are advantageous in terms of their fast response (Shang et al., 2022; Meet Alertgy NICGM, 2022. Blood Glucose Monitoring. In The Convenience of a Wristband. For Diabetics. For Healthcare. Using Non-invasive Deep-Gluco^®^; UBAND-Know Labs, 2021. Bio-RFID™. Transforming Non-Invasive Medical Diagnostics). However, they are not highly sensitive systems, and need to take into account a large number of external factors (such as temperature variability, the effect of pressure and the effect of sweat) to accurately determine the effective permittivity of glucose.

Indigo Diabetes, 2021 (INDIGO DIABETES N.V., Belgium) is a subcutaneous implant which exploit NanoPhotonics Technology (NPT). Indigo Diabetes may work through an on-chip inert and miniature spectrometer by measuring the absorption of light in the individual’s interstitial fluid and by quantifying the concentration of multiple metabolites simultaneously without the use of enzymes or fluorophores (INDIGO Giving people with diabetes the extra sense for health).

Regarding urine test strips, although results are quickly displayed, they are not extremely accurate, since there is an uncertain correlation between glucose in blood and urine; moreover, this technique is not adapt to continuous fast measurements (Oliver et al., 2009; Dziergowska et al., 2019).

Skin Suction Blister (SSB) is a technique based on the accumulation and collection of analytes from interstitial fluid (ISF) by applying vacuum to a skin area. The pressure splits the dermis and epidermis, and the blister fluid from the nearby tissues is next extracted by a syringe (Niedzwiecki et al., 2018). Compared to other ISF sampling approaches, such as RI technology, suction blister sampling is more user-friendly since it is a painless procedure but needs to be optimized for the high sensitivity to temperature and pressure variations and to the interference of other compounds (Oliver et al., 2009; Dziergowska et al., 2019).

Exhaled Breath Analysis (EBA) is an emergent methodology for glycaemia estimation: the exhaled breath gases contains several body metabolism biomarkers, such as acetone. Ketone Breathalyzer (KB) could be a possible device to use in blood glucose level (BGL) estimation, if the correlation between blood glucose and breath acetone levels will be better understood and if the sensitivity to variations of temperature could be decreased or corrected. Previous tests demonstrated 1) the possibility to have good correlation between blood glucose and breath acetone levels and 2) the “Keto Diet” should not have significant impact in terms of accuracy standpoint. Ketone Breathalyzer is cheap, fast and user-friendly; however, to date, EBA remains unreliable and challenging because of the huge mixture of volatile organic compounds (more than 3,500) present in the breath (Bolla and Priefer, 2020; Shokrekhodaei and Quinones, 2020; Dixit et al., 2021). The main advantages and drawbacks of each glucose test technology are listed in Supplementary Table S1, while most of the BGL technologies and devices are summarized in Supplementary Table S2 and in the recent review article by David C. Klonoff group. (Shang et al., 2022).

## 3 Raman

It has been over 24 years since James Lambert, Michael Storrie-Lombardi and Mark Borchert at the Jet Propulsion laboratory, California institute of technology (Caltech), United States, did the first measurement of physiological glucose level using Raman Spectroscopy (RS) (Lambert et al., 1998). They combined this technique with multivariate analysis to measure glucose level in the aqueous humour of a rabbit model. The foremost (most intense) Raman peaks at 911 cm^−1^ and 1,125 cm^−1^ are considered the Raman fingerprints of glucose. The concept of RS would be elegantly developed later for the analysis of other analytes, such as lactate, urea and ascorbate. In 2005, Lambert et al. settled key parameters (laser power, of the order of mW, retinal power density, mW/cm^2^, and exposure time, seconds) to achieve, by RS instruments, a reliable aqueous-humour glucose-level estimation within an acceptable range of safety and patient tolerability (see setup instrument Table 1 row B) (Lambert et al., 2005).

**TABLE 1:** Example of Raman setups.

	Raman excitation source	Beam power (mW)	Spot dimension	Spectral range	Measurement time	Sensitivity, selectivity, accuracy	Spectral data processing	References
A	830 nm diode laser	300	1 mm^2^ (area; on human skin)	355–1,545 cm^−1^	2 s integration time (90 times)	MAE: 5.0%, R^2^: 0.93	PLS analysis, Savitsky – Golay algorithm, EGA Analysis	Enejder et al. (2005)
B	785 nm Ti:sapphire laser	100	∼1.01 mm (diameter; on rabbit retina)	300–1,500 cm^−1^	3 s integration time (50 times)	RMSECV: 24.0mg/dl, R^2^: 0.99	PLS analysis, EGA Analysis	Lambert et al. (2005)
C	785 nm diode laser	15	∼0.002 mm (diameter; on mouse skin)	500–1800 cm^−1^	15 s integration time (25 times)	MAE: 5.7%, R^2^: 0.91	PLS analysis	Shao et al. (2012)
D	785 nm diode laser	400	∼ 8 mm (diameter; on human skin)	541–1818 cm^−1^	10 s integration time (10 times)	R^2^: 0.83	PLS analysis, EMSC, EGA Analysis	Scholtes-Timmerman et al. (2014)
E	830 nm continuous-wave diode laser	300	0.005 mm^2^ (area; on human skin)	300–1800 cm^−1^	3 min integration time (1 time)	ISUP: 1.9 MARD: 25.8%, R^2^: 0.69	ISUP parameter, 15-point Savitsky-Golay 1^st^ order algorithm, EMSC	Lundsgaard-Nielsen et al. (2018)
F	830 nm diode laser (incidence angle of 60°)	250	∼1.6 mm^2^ (area; on swine ear skin)	810–1,650 cm^−1^	4.75 min integration time (1 time)	Intrasubject R^2^: 0.94 Intersubject R^2^: 0.62	Savitzky-Golay filtering, polynomial baseline subtraction, MLR analysis	Kang et al. (2020)
G	830 nm diode laser	50	0.5 mm (diameter; on human skin)	400–1800 cm^−1^	0.1 s integration time (6,000 times)	-	Estimation of power spectral density, Savitzky-Golay filtering	Wróbel et al. (2021)

PLS, partial least squares regression; EGA, clarke error grid; EMSC, extended multiplicative scatter correction; ISUP, Inter-subject unified performance; MAE, mean absolute error; MARD, mean absolute relative difference; MLR, multiple linear regression; RMSECV, root-mean-squared-error of cross validation.

The same year, the first non-invasive (‘‘transcutaneous’’) Raman measurement was made (see setup instrument Table 1 row A). The raw Raman spectra from 17 subjects were dominated by spectral components of the human skin, such as human callus skin, collagen I and III, dermal and epidermal structural proteins, and triolein, as well as of human haemoglobin, water, cholesterol, elastin, phosphatidylcholine and actin, rather than glucose. Consequently, a reliable multivariate calibration method, such as partial least squares (PLS) regression analysis, was employed for the first time in order to analyse the Raman spectra, highlighting the glucose Raman peaks from the other signals (treated as background). In particular, the background was removed by least-squares fitting each spectrum to a fifth order polynomial and subtracting this polynomial from the spectrum. The authors of the study were the first to provide quantitative information about glucose by examining blood samples (Enejder et al., 2005).

Starting from that year, advanced data processing and analysis techniques, allowing the quantitative glucose detection in the combined Raman spectrum, have always been essential. Furthermore, several problems, such as transferring calibration models between patients due to changes in skin compositions, to turbidity-induced alterations in the Raman spectral intensities and peak widths, to the physiological lag between glucose level in blood and interstitial fluid (ISF) as well as to the millimolar detection limit of spontaneous Raman scattering combined with the low physiological concentration of glucose (4–10 mM), made of utmost importance the development of an accurate and robust chemometric (estimating the concentration) algorithm to be used in multiple human subjects or in the same subject at different times. The most commonly methods employed to build a multivariate calibration model were multiple linear regression (MLR), principal component regression (PCR) and partial least squares (PLS); however, they provided reasonable results on single subjects but they had poor predictivity across a cohort of people. Consequently, Barman et al. (2010a) proposed the first nonlinear calibration method for BGM. Construction of nonlinear calibration models has formed a cornerstone of the research efforts, enhancing the glucose prediction performance by almost 30% when compared to PLS-derived results (Barman et al., 2010b; 2010a).

In the same year, Chaiken and co-workers proposed the incorporation into the measurement device of an active tissue placement interface, useful also for modulating the relative optical coupling of mobile tissue components (blood, interstitial fluids) with respect to the one of static components, by impacting on blood circulation (Chaiken et al., 2010).

Dingari et al., in 2011, employed tissue phantom, animal and human subject experimental cases to investigate the impact of other analytes in the blood–tissue matrix, possibly with concentration changing with time, on the measures of BGL using RS (Dingari et al., 2011). Indeed, time-dependent physiological processes make the relation between glucose concentration and spectral data very complex. Amongst other results, these studies reported that Raman-spectra based calibration models were significantly more robust than similar models constructed from NIR absorption spectra when the concentration of the glucose was intentionally kept constant and the concentrations of other spectral interferents were varied randomly. In the same year, the same group analysed algorithms for determining optical properties of tissues using detection of diffuse light scattering; these can be used to correct the impact of a turbid medium, like skin, on Raman scattering signals collected simultaneously (Barman et al., 2011; Wróbel et al., 2021).

In 2012, Shao et al. proposed focusing lasers directly on dermal blood vessels (see Table 1 row C) in order to decrease the background signal, and using Raman measurement sequence within two physical state with and without occlusion of blood circulation in the measured region (Shao et al., 2012). While in the unpressed state, the laser irradiates the “free finger”, thus the Raman signals come from both skin and blood, in the pressed state the measured Raman spectrum included contributions especially from skin constituents, since the pressure placed on the finger triggers the expulsion of most of the blood from the capillary bed. In this last condition, the measured Raman spectrum tends to show lower peaks, including from glucose signal. The most intense (best SNR) characteristic glucose Raman peak appears at 1,125 cm^−1^ and it was used to determine the glucose concentrations both in 1–400 mmol/dl glucose water solution, *in vitro*, and *in vivo* (mouse ear blood vessel). Although the authors state that they could not detect Raman signals at glucose concentrations below 500 mmol/L in water solution, even with very long exposure (10 min), they also state to have observed a clear linear relationship between Raman intensity and blood glucose concentration between ∼50 and ∼160 mmol/L *in vitro* and *in vivo*, after normalization of the glucose peak at 1,125 cm^−1^ using the haemoglobin one at 1,549 cm^−1^ (used as internal standard).

Scholtes-Timmerman group devised in 2014 a new Raman spectroscopic analysis system based on the subtraction of non-glucose-induced variable components from the spectra collected from a cohort of 111 hospitalized patients; the spectra were collected through the forearm skin of the patients, and the variable components where calculated starting from a principal component decomposition on spectra collected from patients grouped according to their glycaemia (see Table 1 row D for some information on the used setup) (Scholtes-Timmerman et al., 2014).

A radically different advancement was proposed in 2016 by the Polish M.S. Wróbel group, which suggested several promising improvement for a miniaturized Raman spectroscopy device: 1) a set-up simplification, in which only the Raman signals at the selected energies are detected by a set of photodetectors; 2) an analysis with Principal Component Analysis (PCA) as explorative analytical method, and a PLS-Regression or other advanced techniques (PLS-DA, SVM-regression) as more precise methods; 3) the detection of Raman signal from the dynamic components in transcutaneous spectroscopy, i.e., the moving blood (Wróbel, 2016). This can be done by exploiting the heartbeat, which modulates the quantity of blood present in the volume of observation. The detection of the dynamic component of the Raman signal varying with the heartbeat should eliminate the signals arising from the static components, such as solid tissues (skin, muscle, vessel walls, lipids, etc.). Steps toward the detection of the pulse frequency and the filtering of the periodical signal with suitable algorithms has further opened the door for the estimation of the pulse-correlated Raman signal by a Raman spectrometer equipped with a photopletysmographic detector (see Table 1 row G). There are several factors that may interfere in the final Raman spectra, such as turbidity-induced variations, changes in fluorescence of blood and/or of bulk tissue, red blood cells aggregation-disaggregation in capillaries, the differential nature of oxy- and deoxy-hemoglobin spectra or the hemodynamic pressure that induces expansion of the vessels; however, this approach has two main advantages. First, the achieved Raman spectra are less patient-specific, because optical parameters and chemical composition of blood are much more consistent over the human population of different races and ages than the ones of the surrounding solid tissue; second, measurements can be performed for a longer time utilizing natural blood pulse, with respect to techniques exploiting external occlusion of the blood flux, assuring better averaging and noise reduction.

An intriguing advance, proposed by Motz et al. (2004) and next developed by Kong et al. (2011), Ghenuche et al. (2012) and Pandey et al. (2017), concerns the development of tailored optical fiber probes for delivery and collection of light to and from the tissue interface. The ultimate achievement on optical fiber-probe based Raman instrument lead to measure reliably the Raman signal from a tissue spot in a lighted room (Motz et al., 2004; Kong et al., 2011; Ghenuche et al., 2012; Pandey et al., 2017).

Lundsgaard-Nielsen et al. (2018) introduced a new RS study of glucose sensing for non-invasive glucose detection in human skin (Lundsgaard-Nielsen et al., 2018). They employed a new table-top confocal Raman spectrometer on a cohort of 35 patients, obtaining highly correlated values between the measured glucose concentrations against the reference ones (see setup in Table 1 row E). The high reliability and accuracy of their device essentially come from two smart developed approaches. First, they presented the evidence that the optimal glucose-containing layer of the skin is below the stratum corneum: collecting the Raman signal from the interstitial fluid compartment at 250–300 μm of depth, they were able to note that the time lag (5 min) of changes in glucose concentration between the capillary and interstitial compartments was clearly lower than others reported in literature (almost 45 min). Moreover, Lundsgaard-Nielsen’s group focused on the development of new spectral data processing: after spectra smoothing and corrections (15-point Savitsky-Golay 1^st^ order algorithm and extended multiplicative scatter correction), they used PLS regression calibrated on 25 days for testing the setup in the last 5 days of the trial. Owing to the different behaviour amongst the PWDs concerning the quantification of BGL, they created, and relied on, an Inter-Subject Unified Performance (ISUP) parameter for comparing results amongst different patients, especially for understanding the optimum detection depth in human skin. The developed device is a small, user-friendly, but non-continuous, Raman-based glucometer, called GlucoBeam, which can operate for ∼10 days without recalibration (Shang et al., 2022). Both calibration models and performance of GlucoBeam system were checked and assessed as comparable to the earlier scanning CGM FreeStyle Libre (Abbott Diabetes Care) system by a very recent report, performing the research on a cohort of 15 insulin-treated subjects with type 1 diabetes (Pleus et al., 2021). However, to date, this Danish device is still under development, probably to solve the need of continuous recalibration and/or for optimizing the reliability of the device.

In 2019, Li et al. employed another approach based on the Renishaw inVia confocal Raman spectrometer, enhanced by Optical Coherence Tomography (OCT) (Li et al., 2019). The measurement was performed by focusing the laser on the microvessels in the superficial layer of the extremely thin nailfold at 100–200 μm depths in the skin, reaching the stratum corneum and epidermis in order to avoid the background arising from the dermis and the physiological lag between blood glucose and ISF glucose. This approach, together with the use of sophisticated mathematical analyses based on PCA combined with back propagation artificial neural network (BP-ANN), enabled to establish high prediction accuracy for BGL estimation.

A radically different advancement was established in 2020 by Kang et al. by the construction of a high optical throughput noncontact vertical Raman system, equipped with an oblique angle (off-axis) laser, which overcome the result of a conventional endoscope-type Raman probe contacted to skin (Singh et al., 2018) and the limits of a conventional radiant angle (on-axis) Raman laser (see setup instrument Table 1 row F) (Kang et al., 2020). While this latter has a limited sampling volume and presses the skin during long measurements, the new oblique angle incidence device maximizes the effective sampling volume since more volume contribute to Raman scattering under the oblique angle illumination, decreases the collection of background signals and allows a stable long-term measurement without skin contact. Furthermore, this new Raman technology, combined with a robust spectral data processing method, allowed to achieve surprising results in terms of linearity between intensities of the glucose Raman peaks (911, 1,060, and 1,125 cm^−1^ glucose fingerprint peaks) and the reference glucose concentrations, especially when an additional Raman band at 1,450 cm^−1^, arising from skin proteins and lipids, was considered for normalization.

## 4 Discussion

In this review, we elucidated the several available approaches for BGL assessment. Choosing the best technique requires to consider a number of factors crucial to its implementation, including invasiveness, continuity, long-term stability, reliability, and miniaturization. Indeed, the ideal glycaemia biosensor should be: 1) quantitative and accurate, at least within the glycaemia ranges discussed in the introduction; 2) reliable, and in particular it should give the value of BGL without delay; 3) cheap, small, and wearable, so that every diabetic patient can have one with or on them all the time; 4) user-friendly: it should be easy to be applied to patients; 5) non-invasive, i.e. it should not need anything inserted in or extracted from the body of the patients; 6) safe and not discomforting, e.g. not causing skin irritation; 7) continuous, or at least able to perform a measurement many times in an hour; 8) exempt from recalibration; this last point, actually, is related to several of the previous ones: (re)calibration is not user-friendly, needs another possibly invasive method for determining BGL, if needed it means that the sensor loses accuracy with time, and measurements are stopped during recalibration.

Regarding (ii), biofluids different from blood or interstitial fluid, like sweat, saliva, exhaled breath condensate, urine and tears, have been considered, since they can be obtained easily (Tang et al., 2020). However, 1) the biofluids glucose concentration is lower than that in blood (see concentrations in physiological fluids of healthy and diabetic patients in (Mohamad Yunos and Nordin, 2020)), therefore more sensitive techniques and a correlation calculation are required; 2) there is a delay in transmission of glucose levels from the blood to the other fluids, and this can represent a problem especially when BGL is rapidly changing, which is maybe the situation in which knowing the BGL and its trajectory is most needed.; 3) daily biofluids collection is sometimes inconvenient or not feasible, especially for elderly patients: e.g., urine cannot be used for continuous glucose-monitoring due to its intermittent nature, or sweat quantities are changing with the environmental situation or the activity of the PWD and there could be the need to extract it with electrophoresis or sonophoresis techniques; 4) the accuracy of the tests is significantly affected by biological parameters, including pH, temperature, mechanical/friction effects, and contamination. More research needs to be address to have an accurate relationship between glucose levels in blood and other fluids before they can be used with therapeutic intervention. As an example, glucose levels in sweat can be correlated, but not perfectly, with blood glucose levels although it lags by about 8 min the levels in blood. (Mohamad Yunos and Nordin, 2020). This did not stop researches for monitoring sweat glucose: we report wearable skin pads based on colorimetric fluorescent probes, a new user-friendly electrochemical sensor that however needs personalized precalibration; a self-powered smartwatch; and the already cited SERS-based wearable sensors (Zhao et al., 2019; Cui et al., 2020; Wang Y. et al., 2021, 2022; Sempionatto et al., 2021).

Minimally invasive devices (i.e. subcutaneous electrochemical sensors) partially decrease the discomfort of fingerstick testing, and are at the moment the most used ones for a continuous glucose monitoring (e.g., the Free Style Libre of Abbott Ltd., United States). However, a shift toward fully non-invasive prototypes would address several limitations of this category.

As stated in the introduction, the best (and maybe only) choice for truly non-invasive continuous blood glucose monitoring devices should be based on measurement where both excitation source and signal can pass through the patients’ tissues, thus probably leaving only electromagnetic techniques and measurements of movements and/or sound/pressure. The developed medical devices should be based on glucose intrinsic molecular properties, such as its near-infrared or mid-infrared absorption coefficient, optical rotation, Raman shifts and photoacoustic properties, and these would allow in principle a direct BGL estimation. However, on humans it is difficult to obtain the best geometry for exploiting quantitatively many of these techniques, or (equivalently) to find a way for normalizing the glucose signal(s); moreover, the signals arising from other, possibly much more abundant, body components may affect the results, and these often depend on characteristics and physiological state of the subjects. Differently than for these devices based on direct measurements on the glucose molecules, indirect and nonspecific methods exploit the effect of glycaemia on the biological/chemical/physical properties of blood and tissues. Indeed, the effects of changing glycaemia on the various signals (e.g., spectra) could arise not only from the different impact of the ones arising from the glucose molecules, but also from variations in the relative abundance or from different chemical modifications of other moieties, e.g. the ones involved in cell metabolism. Of course, both factors (glucose fingerprints and indirect glycaemia-induced variations) are usually present at the same time, and other factors can similarly affect the signal, like changing device properties (sensor–skin interface, source fluctuation in terms of power or spectral characteristic, etc.) or differences due to people’s intrinsic factors, both constant or slowly changing (age, gender, ethnicity, lifestyle, comorbidities, etc.), or fluctuating through the day (activity, hydration, blood supply and pressure, metabolic rate, environmental impacts, etc.).

All these factors must be considered in analysing the data: the spurious signals are often fully or at least partially eliminated through a calibration process, sometimes exploiting the fact that usually glycaemia is assumed to be more fluctuating than what is causing them. Many data analyses exploited advanced analysis techniques, often based on “artificial intelligence”/“machine learning”, for separating the contributions in signals linked to BGL from the spurious ones. In these cases, the system requires a (possibly long) period of calibration in which the analysis algorithm is “trained” by correlating the measured signal/spectrum with the glycaemia measured by a standard method (e.g. finger pricking). A thorough discussion on the details of all the analysis algorithms is beyond the scope of this review; however, in general, the acquired signals are usually “fitted” as a (possibly linear) combination of components, and the “weight” of at least one of them is a function (often linear, but not always) of the BGL; the weight(s) of one or more of the other components can be used for normalization (e.g., signals arising from water, or from haemoglobin, etc.), and this can take into account at least the impact of the above mentioned device factors. This process can be carried out explicitly in data analysis, e.g. in methods based on PCA or simply by considering the heights of peaks specific to glucose and to blood or water, or can be hidden in the used algorithm (e.g. in “deep learning” based methods). The purpose of the calibration is to find the best “components” and the relation of the “weights” of these components with BGL. The calibration can be carried out in single individuals (personalized calibration), or on a cohort of people. The first case produces results that are more reliable, but can require long calibration times and recalibration is needed relatively often; in the second case, it is in principle possible to identify and then correct more interfering factors, and this could bring to a “universal” device, without, or with very short, personalized calibration. For this aim, we envision the production and use of libraries of the possible components of “signals,” perhaps stratified for some characteristics like gender, ethnicity, age groups; these libraries could be uploaded in the BGM devices to be used in the data analysis without calibration, or at least as a starting point for the calibration procedure. Despite the possible use of “blind” AI algorithms for extracting BGL from various signals/spectra and for creating these “libraries,” understanding the source of the various components can help in guiding the analysis process or for the pre-treatment of data.

We discussed above the advantages and the drawbacks of most of the technologies considered for continuous non-invasive quantitative glucose sensing. The most-used electromagnetic-radiation-based quantitative setups in laboratories (i.e., spectrophotometers) usually measure the extinction of light in a transmission configuration, with controlled optical path and a reference for the baseline. However, this is difficult to implement in wearable devices, also for the low transmissivity of the tissues in the spectral ranges of characteristic glucose absorption. Therefore, absorption of the tissues is usually measured in a back-scattering geometry, exploiting the random scattering of radiation from tissues, and this make a universal calibration more difficult.

Raman signals, instead, are per se collected at high angles with respect to the direction of the exciting laser, or even in back-scattering geometry. Raman spectra contains fingerprints of glucose and of other substances including water, and this can be used in principle to normalize the spectrum for calculating directly the concentrations. Moreover, by properly choosing the excitation light in the NIR region, both source and signal (Raman Stokes peaks) fall in the tissues transparency window (∼650–1,350 nm), and this allow to reach deeper regions with more abundance of blood or at least of interstitial fluid. One of the most cited drawbacks for Raman spectroscopy is its low cross section, and therefore a weak signal. Increasing laser power can increase the SNR, but can be harmful, and the maximum permissible exposure (MPE) for skin laser irradiation must be considered. As a rule of thumb, at 788 nm the MPE raises (with the fourth root of time) from 3 × 10–2 J/cm^2^ for times less than or equal to 10^−7^ s, up to 3 J/cm^2^ for 10 s, and above this time the intensity must be below 0.3 W/cm^2^ (Maini, 2013). Since the limits are on intensity or energy density, one possibility to increase the SNR is to increase the illuminated and observed area; however, some of the work discussed above considered instead focusing the laser in a region below the first layers of skin, in a region richer in ISF and/or in capillary blood.

In any case, also the Raman signal from the background is weaker, and this helps in obtaining a sufficient SNR, especially by choosing spectral regions where autofluorescence of tissues is low; moreover, glucose spectrum has features in regions where the background spectrum could be very small. Indeed, in some of the studies reviewed in §2.3 and §3, the height of just one or some of the glucose-fingerprint peaks are correlated to glycaemia (Shao et al., 2012; Kang et al., 2020). For example, in the above cited work by Shao et al. (Shao et al., 2012)., they correlated the height of the peak at 1,125 cm^−1^ with the glycaemia, normalizing the data using a characteristic peak of haemoglobin at 1,549 cm^−1^. It is interesting to notice, however, that the height of the peak at 1,125 cm^−1^ did not tend to zero at very low BGLs (and this can explain why the authors could correlate his intensity to values below the minimum one at which they could discern, with the same setup, Raman spectra of glucose dissolved in water). The remaining non-negligible signal at very low glucose concentration in blood can have two components: 1) close Raman signals from other substances, e.g. the peak at 1,126 cm^−1^ due to a pyrrole half-ring stretching mode in haemoglobin; 2) Raman signal from glucose inside the cells, especially in non-circulating ones.

These two kinds of interference have to be considered in general. The first one can be solved by considering whole spectra and whole spectral components, as discussed above; this can also improve the uncertainty in the final measurement. Indeed, most of the devices being considered for commercialization use this method.

The second effect is subtler. Although the total glucose in the observation volume (the one from which the signal is collected) is correlated with the concentration of glucose in plasma, being able to discern the signal arising only from mobile fluids would allow a direct determination of the glycaemia, independent from or much less dependent on calibrations and corrections. As discussed above, one possibility is to consider the spectral differences in the same tissue when there are different amounts of liquids. This has been done, e.g., with occlusion spectroscopy, but maybe the most promising approach for continuous wearable devices is to exploit the differences in blood and interstitial fluid content caused by the heart pulse. It could be possible to consider just the periodically varying part in continuously-collected Raman signals, but extracting the changing component could be helped by considering the correlation between the cross correlation of the heartbeat, measured e.g. by gravitometry, pressure sensors, or PPG signal, with the Raman one. Data analysis should also consider that the spectral changes do not arise simply by increasing blood content in the tissue: the observation volume is approximately constant, so the amount of observed blood anticorrelates with the observed quantity of the remaining tissues, and these contain glucose, water, and other biomolecules as well. The idea of having other sensors integrated in a single device is interesting also because collecting other parameters together with the heartbeat (like temperature, blood pressure, blood oxygen level, etc.) can help analysing the spectra and correcting the measured glycaemia: e.g., it could be possible to correlate some “signal components” changes to some of these parameters, and this will help in determining their weight in the total detected spectra.

## 5 Conclusion and perspectives

A fully integrated and self-powered NIGM smartwatch or similar device is highly desirable in daily diabetes management. Several findings on the technical aspects of NIGM devices have matured over the last decades. In particular, Raman technology, together with sophisticated prediction algorithms, seems to have solid basis for further improvements in this field. As an example, the Raman-based GlucoBeam (RSP System A/S, Denmark) seems one of the best user-friendly device to provide self-monitoring glucose measurement, although it still suffers from poor reliability, poor long-term stability and unsolved issues with variations amongst patients (Lundsgaard-Nielsen et al., 2018; Pleus et al., 2021).

In order to expand the use of RS methodology, future efforts should aim at 1) maximizing the sensitivity in experiments 2) reducing the integration time (or exploiting changes in time of the signal) 3) miniaturizing the system and 4) improving the understanding of the factors affecting the inter-individual variations. Notably, we believe that a real-time and continuous RS device exploiting blood pulse (combined with the suitable detection algorithm) represents nowadays the best emergent technology for our purpose, even if the reliability of such device should be verified during both storing status and dynamic activities.

## Glossary

BGL: Blood Glucose Level
BGM: Blood Glucose Monitoring
Bio-RFID: Body RadioFrequency Identification
BP-ANN: Back Propagation Artificial Neural Network
BS: Bioimpedance Spectroscopy
CGM: Continuous Glucose Monitoring
EBA: Exhaled Breath Analysis
EDT: Enzymatic Detection Technology
EGA: Clarke Error Grid
EMS: ElectroMagnetic Sensing
EMSC: Extended Multiplicative Scatter Correction
FDA: Food and Drug Administration
FIR: Far Infrared Radiation
FIRS: Far Infrared Radiation Spectroscopy
FRET: Fluorescence Resonance Energy Transfer
FT: Fluorescence Technology
KB: Ketone Breathalyzer;
ISF: InterStitial Fluid
ISUP: Inter-Subject Unified Performance
MHC: Metabolic Heat Conformation
MLR: Multiple Linear Regression
MI: Minimally invasive
MIRS: Mid Infrared Spectroscopy
MMS: Millimetre and Microwave Sensing
MPE: Maximum Permissible Exposure
MT: MultiTechnologies
N/A: Not Available
NCGM: Non Continuous Glucose Monitoring
NI: Non-invasive
NICBGL: Non-Invasive Continuous Blood Glucose Level
NIRS: Near Infrared Spectroscopy
NPT: NanoPhotonics Technology
OCT: Optical Coherence Tomography
OPT: Optical Polarimetry Technology
OS: Occlusion Spectroscopy
PAS: PhotoAcoustic Spectroscopy
PCA: Principal Component Analysis
PEG: Parkes Error Grid
PLS: Partial Least Squares
PLS-DA: Partial Least Squares-Discriminant Analysis
PPG: PhotoPlethysmoGraphy
PWD: People With Diabetes
QCL: Quantum Cascade Laser;
RFST: RadioFrequency Sensor Technology
RI: Reverse Iontophoresis
RS: Raman Spectroscopy
SNR: Signal to Noise Ratio
SPRT: Surface Plasmon Resonance Technology
SSB: Skin Suction Blister
ST: Sonophoresis Technology
SVM: Support Vector Machine
TOF: Time of Flight
THz-TDS: Terahertz Time-Domain Spectroscopy
UT: Ultrasound Technology
