# Supplementary material for: Is Raman the best strategy towards the development of non-invasive continuous glucose monitoring devices for diabetes management?
Source: Front Chem. 2022 Sep 26;10:994272. doi: 10.3389/fchem.2022.994272 (PMC9548653; doi:10.3389/fchem.2022.994272)
Supplement: Supplementary file 1 [file DataSheet1.pdf]

frontiers

Supplementary Material

Is Raman the best strategy towards the development of non-invasive continuous glucose monitoring devices for diabetes management?

Biagio Todaro[1,*], Filippo Begarani[3,4], Federica Sartori[3,4], and Stefano Luin[1,2,*]
[1]NEST Laboratory, Scuola Normale Superiore, Piazza S. Silvestro 12, I-56127 Pisa, Italy
[2]NEST, Istituto Nanoscienze, CNR, Piazza San Silvestro 12, I-56127 Pisa, Italy
[3]P.B.L. SRL, Via Volta 8, I-43046, Solignano (PR), Italy
[4]Omnidermal Biomedics SRL, Via Volta 8, I-43046, Solignano (PR), Italy
[*]Correspondence: biagio.todaro@sns.it, s.luin@sns.it

Supplementary Table 1. Advantages and drawbacks of techniques feasible for glycaemia sensing

Technique	Advantages	Disadvantages
**Near Infrared (NIR) (Light wavelength 0.7µm-2.5µm)**	• The signal intensity is directly proportional to concentration of the analyte (glucose)<br>• Water transparent band in the NIR region<br>• Relatively low-cost materials needed<br>• Method also works in presence of interfering substances, such as plastic or glass<br>• High sensitivity of photoconductive detectors	• Glucose concentration is too low for accurate detection so complex machine learning model is required for interpretation<br>• High scattering level<br>• Problems of selectivity for separation of glucose from other physiological substances and tissue components (water, haemoglobin, proteins, fat, etc.).<br>• Weak sensitivity and stability<br>• Frequent recalibration
**Mid InfraRed (MIR) (Light wavelength 2.5µm-25µm)**	• Lower scattering and higher absorption compared to NIR<br>• The signals are more specific and better delineated<br>• Glucose can absorb specific MIR wavelengths, thus its concentration can be measured with more accuracy<br>• Possibility to be combined with the measurement of thermal Infrared radiation	• The light has limited penetration in tissue (few micrometres) so only (diffuse) reflection may be feasible<br>• Strong haemoglobin and blood water absorption that interferes with measurement<br>• Expensive equipment
**Far InfraRed (FIR) (Light wavelength 25µm-1000µm)**	• Frequent calibration is not required<br>• Less sensitive towards scattering than NIR and MIR	• The emission radiation intensity depends on temperature and substance thickness<br>• Strong water absorption makes extremely difficult the identification of other analytes, such as glucose, in the sample

Technique	Advantages	Disadvantages
**Time of Flight (TOF) and Terahertz Time-Domain Spectroscopy (THz-TDS)**	• Strong absorption and dispersion for glucose molecule<br>• Immune to background noise<br>• Study of a broad frequency range with a single ultrashort pulse<br>• Complex permittivity measurement with a single scan	• Long measurement time<br>• Low spatial and depth resolution<br>• Expensive and difficult to miniaturize equipment
**Raman Spectroscopy (RS)**	• Chemical stability<br>• Minimally sensitive towards temperature changes and water presence<br>• High specificity<br>• Good penetration depth with NIR sources<br>• Suitable on any substrate (including opaque ones) since it measures scattered light	• The laser radiation can be dangerous during CGM<br>• Unstable laser wavelength and intensity<br>• Long collection time<br>• Low SNR: method is susceptible to noise arising from fluorescence (or possibly Raman) from other molecules.
**Surface-enhanced Raman scattering (SERS)**	• High sensitivity, selectivity and specificity<br>• User-friendly approach (for sensor patches)	• Subcutaneous injection of metal materials can produce toxicity and skin damages<br>• Typically expensive tools
**Millimetre and Microwave sensing (MMS)**	• Deep penetration of signal<br>• No risk for ionization<br>• Highly sensitive for glucose detection	• Poor selectivity<br>• Very much sensitive to physiological parameters such as sweating, breathing and cardiac activity
**Photoacoustic Spectroscopy (PAS)**	• Relatively simple and compact sensor design<br>• Used optical radiation (wavelength from the ultraviolet to the MIR ranges) isn't harmful to skin<br>• Not susceptible to water, NaCl, cholesterol and albumin<br>• PA signal is not influenced by scattering particles	• Signal is susceptible to temperature, motion, pulsation and surrounding acoustic noise, so low SNR<br>• Long integration time.
**Enzymatic detection technology (EDT)**	• Direct and efficient collection in situ	• Minimally invasive<br>• Under development technology

Technique	Advantages	Disadvantages
**Reverse iontophoresis (RI)**	• Electrodes are not difficult to manufacture and be applied to the skin with minimum training.<br>• Good correlation between glucose level in the ISF and in the blood under stable conditions.<br>• Glucose measurement is based on the well-known enzymatic method.	• Skin irritation due to the passage of current.<br>• Susceptible to sweating.<br>• Rapid changes of glucose concentration cannot be detected accurately
**Sonophoresis Technology (ST)**	• User-friendly approach as there is little side-effect to skin<br>• Glucose measurement is based on well-known enzymatic method<br>• Good control on the amount of glucose that can be extracted for the analysis	• Susceptible to temperature and pressure variations, to environmental parameters, and to the presence of other compounds
**Fluorescence Technology (FT)**	• Highly sensitive (glucose concentration as small as 25 μM), allowing even single-molecule detection<br>• High specificity because of distinctive optical properties of molecules<br>• It can measure analyte concentration in terms of fluorescence intensity and decay times<br>• Fluorescence lifetime can be precisely measured in scattering media such as skin layers, indicating that fluorescence technology is suitable for glucose monitoring devices based on transdermal sensing	• Needs an "exogenous" fluorescence-based sensor/indicator in contact with the analyte<br>• Depending on the sensor, can be sensitive to local pH changes and/or oxygen levels<br>• Biocompatibility issues due to local tissue trauma<br>• Potential toxicity issues due to foreign body in biological media<br>• Short lifespan of the fluorophore: limitations associated with photostability and loss of recognition capability<br>• Susceptible to autofluorescence
**Metabolic Heat Conformation (MHC)**	• Physiological parameters for glucose prediction are relatively easy to measure using well established technologies	• Indirect quantification of glucose; needs personalized calibration<br>• Sensitive towards variation in temperature, sweat, other physio-pathological conditions
**Bioimpedance spectroscopy (BS)**	• Cheap method<br>• Suitable for CGM<br>• Easy measurement on the skin	• Sensitive to variations of temperature and motion, to sweat and to water content<br>• Low specificity to glucose<br>• Require long calibration periods

Technique	Advantages	Disadvantages
**Optical Coherence Tomography (OCT)**	• High resolution and good SNR<br>• Not susceptible to blood pressure, haematocrit and cardiac activity<br>• High penetration depth	• Sensitive to temperature changes on the skin and motion<br>• Suffers from tissue inhomogeneity<br>• Lack of chemical specificity
**Optical Polarimetry Technology (OPT)**	• Optical components can be easily miniaturized<br>• The laser intensity variation will not change too much the glucose prediction<br>• Ease of use	• Sensitive to interference from other optically active compounds, temperature, pH changes and motion<br>• Applied in the eye: lag time could be up to 30 min<br>• Requirement of external laser source and proper alignment with eye<br>• A non-contact method needs to be developed for commercial use
**Surface Plasmon Resonance (SPR)**	• Highly sensitive for glucose molecule detection and for small changes of blood glucose concentration<br>• No need for statistical calibration models due to its conventional electrical model nature	• Need contact to the analyte<br>• Long calibration process<br>• Bulky in size<br>• Sensitive to temperature, sweat and motion
**Electromagnetic sensing (EMS)**	• Safe technology for the absence of ionization of other molecules of the body<br>• Low-cost and easily miniaturized	• Lack of selectivity because dielectric constant is affected by other blood components<br>• Sensitive to temperature
**Ultrasound Technology (UT)**	• Well established technology<br>• User-friendly approach since not harmful to tissue cells<br>• High sensitivity due to the long penetration below the skin and other tissues<br>• Immune to skin colour variation<br>• Wide range of frequencies available for use	• Limited accuracy with ultrasound, so often coupled with NIR in a multi-model approach<br>• Expensive<br>• Not suitable for CGM<br>• Pressure changes and temperature fluctuations can cause interferences

Technique	Advantages	Disadvantages
**Occlusion Spectroscopy (OS) – based on R-NIR scattering / extinction**	• Suitable for the non-invasive measurement of arterial glucose	• Susceptibility to many endovascular variables, such as pharmacological treatment, internal erythrocyte aggregation, free fatty acid concentration and chylomicrons (ULDLs)
**RadioFrequency Sensor Technology (RFST)**	• No risk of damaging tissue<br>• Fast response	• Not sensitive nor selective system<br>• Susceptible to temperature, pressure and sweat
**NanoPhotonics Technology (NPT)**	• Highly sensitive and specific	• Sensitive for pH changes and/or oxygen levels<br>• Potential toxicity issues due to foreign body in biological media<br>• Limitations associated with photostability and loss of recognition capability
**Urine test strips**	• Results are quickly displayed	• Readings aren’t extremely accurate<br>• Sample extraction isn’t user-friendly<br>• Need of privacy for sampling<br>• Uncertain correlation between glucose in blood and urine<br>• Not adapt to continuous fast measures.
**Skin suction blister technique (SCB)**	• Well-tolerated<br>• Painless procedure<br>• Low infection risk	• Requires an “external” sensor<br>• Susceptible to temperature and pressure variations and to the presence of other compounds
**Exhaled Breath Analysis (EBA)**	• Ease of use<br>• “Keto Diet” should not have significant impact in terms of accuracy standpoint<br>• Good correlation between blood glucose and breath acetone levels<br>• Not expensive and quick results	• Sensitive to variations of temperature<br>• Clear correlation still needs to be established

Supplementary Table 2. Examples of available, recently-studied, or under-development devices for glycaemia monitoring classified on the basis of the exploited technology.

Technology	Device (Company)	Target	Comment	Status	Ref
NIR Spectroscopy	Combo Glucometer (Cnoga Medical, Israel)	Finger	NI, NCGM. Personalized calibration needed	Available	(Segman, 2018; Villena Gonzales et al., 2019; Shang et al., 2022) (URL: https://www.medicalplasticsnews.com/news/device-for-diabetes-exhibiting-at-medica/)
	- (Tech4Life, US)	Finger	NI, NCGM	Available (trials in several countries)	(Dixit et al., 2021)
	Wizmi^{TM} (Wear2b Ltd., Israel)	Arm wrist	NI, CGM	Proof of concept	(Hadar et al., 2019; Villena Gonzales et al., 2019; Shang et al., 2022)
	LifeLeaf® (LifePlus, US)	Arm wrist	PPG	Under development	(Shang et al., 2022) (URL: https://www.lifeplus.ai)
	- (Polytechnic University of Catalunya, Catalonia)	Finger	PPG	Under development	(Monte-Moreno, 2011; Villena Gonzales et al., 2019)
	- (Karunya University, India)	Finger, forearm	PPG	Under development	(Villena Gonzales et al., 2019)
	NBM-200G (OrSense Ltd., Israel)	Finger	NI	Withdrawn	(Villena Gonzales et al., 2019) (URL: http://www.orsense.com/product.php?ID=49)
	Diasensor 1000 (Biocontrol Technology, US)	Skin	N/A	Withdrawn	(Villena Gonzales et al., 2019)
	HELO Extense (World Global Network, US)	Finger	NI, NCGM, PPG	Never released	(Villena Gonzales et al., 2019) (URL: https://website.worldgn.com/heloextense/)
	- (TouchTrack Pro)	N/A	N/A	Never released	(Villena Gonzales et al., 2019)
	GluControl® GC300 (Samsung Fine Chemicals Co., Ltd. & Arith. Med Gmb. H, US)	N/A	N/A	Never released	(Villena Gonzales et al., 2019)
MIR Spectroscopy	Diamontech D-Band (DiaMonTech AG, Germany)	Finger	NI, NCGM	Under development	(Shang et al., 2022; DiaMonTech: Non-Invasive Blood Glucose Monitoring.) (URL: https://www.diamontech.de/home#technology)
	Diamontech D-Pocket (DiaMonTech AG, Germany)	Finger	NI, NCGM	Under development	(Shang et al., 2022; DiaMonTech: Non-Invasive Blood Glucose Monitoring.) (URL: https://www.diamontech.de/home#technology)

Technology	Device (Company)	Target	Comment	Status	Ref
	Diamontech D-Base (DiaMonTech AG, Germany)	Arm wrist	MI, CGM, “photothermal detection”	Under development	(Shang et al., 2022; DiaMonTech: Non-Invasive Blood Glucose Monitoring.) (URL: https://www.diamontech.de/home#technology)
	- (Tohoku University, Japan)	Oral mucosa, Inner lips	Trapezoidal multireflection	Under development	(Kino et al., 2016; Villena Gonzales et al., 2019)
	- (Swiss Federal Institute of Technology, Switzerland)	Forearm	Photoacoustic detection, QCLs	Under development	(Kottmann et al., 2016; Villena Gonzales et al., 2019)
Raman Spectroscopy (RS)	GlucoBeam (RSP System A/S, Denmark)	Hand palm	NI, NCGM, Accuracy affected by time-lag	Available (under development)	(Lundsgaard-Nielsen et al., 2018; Villena Gonzales et al., 2019; Shang et al., 2022) (URL: https://rspsystems.com/glucobeam/)
	C8 Medisensors (C8 Medisensors inc., US)	Skin	Needed an additional influx of capital to finalize the design	Never released	(Villena Gonzales et al., 2019) (URL: https://www.medgadget.com/2012/10/c8-non-invasive-optical-glucose-monitor-system-cleared-for-sale-in-europe-video.html)
Photoacoustic Spectroscopy (PAS)	- (Electronics and Telecomm. Research Inst. of Korea, Republic of Korea)	Fingertip	NI, NCGM	Under development	(Sim et al., 2018; Villena Gonzales et al., 2019)
	Aprise (Glucon, US)	Upper arm	NI, CGM	Never released	(Villena Gonzales et al., 2019) (URL: https://www.diabetesincontrol.com/continuous-non-invasive-glucose-monitoring-device-shows-positive-results-in-cl/)
Reverse Iontophoresis (RI)	GluCall (K.M.H Co., Ltd., South Korea)	Arm wrist	MI, CGM	Available (Under development)	(Villena Gonzales et al., 2019) (URL: https://kmholding.en.ec21.com/Glucall_Non-Invasive_Glucose_Monitor--969741_969746.html)
	SugarBEAT (Nemaura Medical, United Kingdom)	Upper arm	MI, CGM (daily disposable Sensor and Transmitter)	Available	(Villena Gonzales et al., 2019; Shang et al., 2022) (URL: https://nemauramedical.com/nmrd-info/)
	- (University of Bath, United Kingdom)	Skin	MI, CGM graphene-based transdermal platform	Under development	(Lipani et al., 2018; Villena Gonzales et al., 2019)
	GlucoWatch (Cygnus Inc., US)	Arm wrist	Reliability problems	Withdrawn	(Villena Gonzales et al., 2019; Shang et al., 2022) (URL: http://www.mendosa.com/glucowatch.htm)

Technology	Device (Company)	Target	Comment	Status	Ref
Fluorescence	Eversense® (Senseonics, US)	Upper arm	MI, CGM Underskin sensor, for 90 days	Available	(Villena Gonzales et al., 2019; Jafri et al., 2020; Shang et al., 2022) (URL: https://global.eversensediabetes.com)
	- (Profusa, Inc., US)	Skin	MI, CGM, placed under the skin	Under development	(Gamsey et al., 2006; Villena Gonzales et al., 2019)
	DermalAbyss (Massachusetts Institute of Technology, US)	Skin	NI, CGM	Under development	(Vega et al., 2017; Villena Gonzales et al., 2019) (URL: https://www.dezeen.com/2017/06/01/mit-researchers-tattoo-inks-act-health-trackers-design-technology/)
	- (University of Western Ontario, Canada)	Tears	MI, CGM, FRET	Under development	(Chen et al., 2017; Villena Gonzales et al., 2019)
Metabolic Heat Conformation (MHC)	GlucoGenius (ESER Health Care Digital Technology Co Ltd, Taiwan)	Finger	NI, NCGM	Available	(Villena Gonzales et al., 2019) (URL: https://www.computex.biz/eserhealth/default.aspx?ContentTab=Video&vid=166&t=specific)
	- (Health-Care Computer, Japan)	Finger	NI, NCGM	Under development	(Okura et al., 2018; Villena Gonzales et al., 2019)
	G2 Mobile (Eser Digital, India)	Finger	NI, NCGM	Under development	(Villena Gonzales et al., 2019) (URL: https://www.desertcart.in/products/148404256-eser-non-invasive-prickless-bloodless-glucose-meter-blood-sugar-test-no-strip-no-needles)
	- (Hitachi Ltd., Japan)	Finger	N/A	Never released	(Villena Gonzales et al., 2019)
Bioimpedance spectroscopy (BS)	Glucoband (Calisto Medical, Inc., US)	Arm wrist	MI, CMG	Withdrawn	(Villena Gonzales et al., 2019) (URL: https://www.medgadget.com/2005/06/glucoband.html)
	Pendra (Pendragon Medical Ltd, Switzerland)	Arm wrist	Poor accuracy	Withdrawn	(Villena Gonzales et al., 2019)
Millimetre and Microwave sensing (MMS)	GlucoWise (MediWise, United Kingdom)	Hand	NI, NCGM	Available (not for public)	(Villena Gonzales et al., 2019; Shang et al., 2022) (URL: https://gluco-wise.com)
	- (University of Waterloo and Google, US)	N/A	NI	Under development	(Shaker et al., 2018; Villena Gonzales et al., 2019)
	- (University of Cardiff, United Kingdom)	Abdomen	NI, Microwave Split-ring resonance	Under development	(Choi et al., 2017; Villena Gonzales et al., 2019)
	- (Caltech University, US)	Ear lobe	NI, NCGM	Under development	(Villena Gonzales et al., 2019)

Technology	Device (Company)	Target	Comment	Status	Ref
	- (University of Erlangen-Nuremberg, Germany)	Skin	NI, NCGM	Under development	(Villena Gonzales et al., 2019)
Sonophoresis	Symphony (Echo Therapeutics, US)	Skin	MI, CGM	Under development	(Villena Gonzales et al., 2019) (URL: https://www.huffpost.com/entry/glucose-monitoring_b_1503881)
Enzymatic detection	Guardian Sensor 3 (Medtronic Plc., US)	Abdomen	MI, NCGM	Available	(Cappon et al., 2017; Christiansen et al., 2017; Lee et al., 2021; Shang et al., 2022) (URL: https://news.medtronic.com/MiniMed-R-670G-Hybrid-Closed-Loop-System)
	Dexcom G6 (DexCom, Inc., US)	Abdomen	MI, NCGM	Available	(Cappon et al., 2017; Boscari et al., 2021, 2022; Lee et al., 2021; Shang et al., 2022) (URL: https://www.dexcom.com)
	Free Style Libre (Abbott Ltd., US)	Upper arm	MI, NCGM	Available	(Cappon et al., 2017; Blum, 2018; Galindo et al., 2020; Jafri et al., 2020; Lee et al., 2021; Shang et al., 2022) (URL: https://www.medicaldevice-network.com/projects/freestyle-libre/)
	K'Watch (PKvitality, France)	Arm wrist	MI, CGM Skin patch	Available (under development)	(Villena Gonzales et al., 2019; Shang et al., 2022) (URL: https://www.pkvitality.com)
	- (Ulsan National Inst. of Science and Technology, South Korea)	Tears	MI, CGM	Only research paper	(Park et al., 2018; Villena Gonzales et al., 2019)
	The biosensor platform (The IQ Global Group Ltd., Australia)	Saliva	MI, CGM, Organic Transistor	Under development	(Galindo et al., 2020) (URL: https://www.medicaldesignandoutsourcing.com/harvards-wyss-institute-wants-to-create-a-covid-19-antibody-test-strip/)
	Mouthguard glucose sensor (Tokyo Medical and Dental University, Japan)	Saliva	MI, CGM	Only research paper	(Arakawa et al., 2020)
	NovioSense (Novio Tech Campus, Netherlands)	Tears	MI, CGM	Under development (Only for type 1 diabetes)	(Kownacka et al., 2018; Shang et al., 2022)
	- (KTH Royal Inst. of Technology, Sweden)	Forearm	MI, CGM	Under development	(Ribet et al., 2018; Villena Gonzales et al., 2019)
Optical Coherence Tomography (OCT)	- (National Cheng Kung University, Taiwan)	Fingertip	NI, NCGM	Under development	(Chen et al., 2018; Villena Gonzales et al., 2019)

Technology	Device (Company)	Target	Comment	Status	Ref
Combination of:<br>•Ultrasound<br>•Thermal<br>•Electromagnetic sensing	GlucoTrack (Integrity Applications, Israel)	Ear lobe	NI, NCGM individually calibrated Personal Ear Clip	Available (Not for type 1 diabetes)	(Harman-Boehm et al., 2010; Villena Gonzales et al., 2019; Shang et al., 2022) (URL: https://www.techyv.com/tips/top-10-highly-useful-techs-for-checking-blood-glucose-level/)
	Egm1000™ (Evia Medical Technologies Limited, Saudi Arabia)	Ear lobe	NI, NCGM individually calibrated Personal Ear Clip	Available (Not for type 1 diabetes)	(Mosli and Madani, 2021) (URL: https://pharmatee.com/product/egm-1000/)
RadioFrequency Sensor Technology (RFST)	Alertgy® (Alertgy Ltd., US)	Arm wrist	NI, CGM	Under development	(Meet Alertgy® NICGM. Blood Glucose Monitoring) (URL: https://www.alertgy.com/technology/)
	UBAND™ (Know Labs, US)	Arm wrist	NI, CGM, Bio-RFID	Under development	(Shang et al., 2022; UBAND-Know Labs. Bio-RFID™) (URL: https://www.businesswire.com/news/home/20191121005261/en/Know-Labs-Unveils-New-UBAND-Design-and-Smartphone-App)
NanoPhotonics Technology (NPT)	Indigo Diabetes (INDIGO DIABETES N.V., Belgium)	Skin	MI, CGM, placed under the skin	Proof of concept	(INDIGO Giving people with diabetes the extra sense for health) (URL: https://indigomed.com/product/)

Ref: Reference(s); MI: Minimally invasive, NI: Non-invasive, NCGM: Non continuous glucose monitoring, CGM: Continuous glucose monitoring, QCL: Quantum Cascade lasers, PPG: Photoplethysmography. Bio-RFID: Body Radio Frequency Identification. FRET: Fluorescence Resonance Energy Transfer. PEG: Parkes Error Grid, EGA: Clarke Error Grid, N/A: not available.

References

Arakawa, T., Tomoto, K., Nitta, H., Toma, K., Takeuchi, S., Sekita, T., et al. (2020). A Wearable Cellulose Acetate-Coated Mouthguard Biosensor for In Vivo Salivary Glucose Measurement. *Anal. Chem.* 92, 12201–12207. doi: 10.1021/acs.analchem.0c01201.

Blum, A. (2018). Freestyle Libre Glucose Monitoring System. *Clin. Diabetes* 36, 203–204. doi: 10.2337/cd17-0130.

Boscari, F., Vettoretti, M., Amato, A. M. L., Vallone, V., Uliana, A., Iori, E., et al. (2021). Comparing the accuracy of transcutaneous sensor and 90-day implantable glucose sensor. *Nutr. Metab. Cardiovasc. Dis.* 31, 650–657. doi: 10.1016/j.numecd.2020.09.006.

Boscari, F., Vettoretti, M., Cavallin, F., Amato, A. M. L., Uliana, A., Vallone, V., et al. (2022). Implantable and transcutaneous continuous glucose monitoring system: a randomized cross over trial comparing accuracy, efficacy and acceptance. *J. Endocrinol. Invest.* 45, 115–124. doi: 10.1007/s40618-021-01624-2.

Cappon, G., Acciaroli, G., Vettoretti, M., Facchinetti, A., and Sparacino, G. (2017). Wearable Continuous Glucose Monitoring Sensors: A Revolution in Diabetes Treatment. *Electronics* 6, 65. doi: 10.3390/electronics6030065.

Chen, L., Tse, W. H., Chen, Y., McDonald, M. W., Melling, J., and Zhang, J. (2017). Nanostructured biosensor for detecting glucose in tear by applying fluorescence resonance energy transfer quenching mechanism. *Biosens. Bioelectron.* 91, 393–399. doi: 10.1016/j.bios.2016.12.044.

Chen, T.-L., Lo, Y.-L., Liao, C.-C., and Phan, Q.-H. (2018). Noninvasive measurement of glucose concentration on human fingertip by optical coherence tomography. *J. Biomed. Opt.* 23, 1. doi: 10.1117/1.JBO.23.4.047001.

Choi, H., Luzio, S., Beutler, J., and Porch, A. (2017). Microwave noninvasive blood glucose monitoring sensor: Human clinical trial results. in *2017 IEEE MTT-S International Microwave Symposium (IMS)* (Honololu, HI, USA: IEEE), 876–879. doi: 10.1109/MWSYM.2017.8058721.

Christiansen, M. P., Garg, S. K., Brazg, R., Bode, B. W., Bailey, T. S., Slover, R. H., et al. (2017). Accuracy of a Fourth-Generation Subcutaneous Continuous Glucose Sensor. *Diabetes Technol. Ther.* 19, 446–456. doi: 10.1089/dia.2017.0087.

DiaMonTech: Non-Invasive Blood Glucose Monitoring. Available at: https://www.diamontech.de/home.

Dixit, K., Fardindoost, S., Ravishankara, A., Tasnim, N., and Hoorfar, M. (2021). Exhaled Breath Analysis for Diabetes Diagnosis and Monitoring: Relevance, Challenges and Possibilities. *Biosensors* 11, 476. doi: 10.3390/bios11120476.

Galindo, R. J., Migdal, A. L., Davis, G. M., Urrutia, M. A., Albury, B., Zambrano, C., et al. (2020). Comparison of the FreeStyle Libre Pro Flash Continuous Glucose Monitoring (CGM) System and Point-of-Care Capillary Glucose Testing in Hospitalized Patients With Type 2 Diabetes Treated With Basal-Bolus Insulin Regimen. *Diabetes Care* 43, 2730–2735. doi: 10.2337/dc19-2073.

Gamsey, S., Suri, J. T., Wessling, R. A., and Singaram, B. (2006). Continuous Glucose Detection Using Boronic Acid-Substituted Viologens in Fluorescent Hydrogels: Linker Effects and Extension to Fiber Optics. *Langmuir* 22, 9067–9074. doi: 10.1021/la0617053.

Hadar, E., Chen, R., Toledano, Y., Tenenbaum-Gavish, K., Atzmon, Y., and Hod, M. (2019). Noninvasive, continuous, real-time glucose measurements compared to reference laboratory venous plasma glucose values. *J. Matern. Fetal Neonatal Med.* 32, 3393–3400. doi: 10.1080/14767058.2018.1463987.

Harman-Boehm, I., Gal, A., Raykhman, A. M., Naidis, E., and Mayzel, Y. (2010). Noninvasive Glucose Monitoring: Increasing Accuracy by Combination of Multi-Technology and Multi-Sensors. *J. Diabetes Sci. Technol.* 4, 583–595. doi: 10.1177/193229681000400312.

INDIGO Giving people with diabetes the extra sense for health Available at: https://indigomed.com.

Jafri, R. Z., Balliro, C. A., El-Khatib, F., Maheno, M. M., Hillard, M. A., O’Donovan, A., et al. (2020). A Three-Way Accuracy Comparison of the Dexcom G5, Abbott Freestyle Libre Pro, and Senseonics Eversense Continuous Glucose Monitoring Devices in a Home-Use Study of Subjects with Type 1 Diabetes. *Diabetes Technol. Ther.* 22, 846–852. doi: 10.1089/dia.2019.0449.

Kino, S., Omori, S., Katagiri, T., and Matsuura, Y. (2016). Hollow optical-fiber based infrared spectroscopy for measurement of blood glucose level by using multi-reflection prism. *Biomed. Opt. Express* 7, 701. doi: 10.1364/BOE.7.000701.

Kottmann, J., Rey, J., and Sigrist, M. (2016). Mid-Infrared Photoacoustic Detection of Glucose in Human Skin: Towards Non-Invasive Diagnostics. *Sensors* 16, 1663. doi: 10.3390/s16101663.

Kownacka, A. E., Vegelyte, D., Joosse, M., Anton, N., Toebes, B. J., Lauko, J., et al. (2018). Clinical Evidence for Use of a Noninvasive Biosensor for Tear Glucose as an Alternative to Painful Finger-Prick for Diabetes Management Utilizing a Biopolymer Coating. *Biomacromolecules* 19, 4504–4511. doi: 10.1021/acs.biomac.8b01429.

Lee, I., Probst, D., Klonoff, D., and Sode, K. (2021). Continuous glucose monitoring systems - Current status and future perspectives of the flagship technologies in biosensor research -. *Biosens. Bioelectron.* 181, 113054. doi: 10.1016/j.bios.2021.113054.

Lipani, L., Dupont, B. G. R., Doungmene, F., Marken, F., Tyrrell, R. M., Guy, R. H., et al. (2018). Non-invasive, transdermal, path-selective and specific glucose monitoring via a graphene-based platform. *Nat. Nanotechnol.* 13, 504–511. doi: 10.1038/s41565-018-0112-4.

Lundsgaard-Nielsen, S. M., Pors, A., Banke, S. O., Henriksen, J. E., Hepp, D. K., and Weber, A. (2018). Critical-depth Raman spectroscopy enables home-use non-invasive glucose monitoring. *PLOS ONE* 13, e0197134. doi: 10.1371/journal.pone.0197134.

Meet Alertgy® NICGM. Blood Glucose Monitoring. In The Convenience of a Wristband. For Diabetics. For Healthcare. Using Non-invasive Deep-Gluco® Available at: https://www.alertgy.com.

Monte-Moreno, E. (2011). Non-invasive estimate of blood glucose and blood pressure from a photoplethysmograph by means of machine learning techniques. *Artif. Intell. Med.* 53, 127–138. doi: 10.1016/j.artmed.2011.05.001.

Mosli, H. H., and Madani, B. M. (2021). Performance evaluation of egm1000^{TM} non-invasive glucose monitoring device in patients with type 2 diabetes and subjects with prediabetes. *Int. J. Med. Dev. Ctries.* 5(4), 9. doi: 10.24911/IJMDC.51-1611360395.

Okura, T., Teramoto, K., Koshitani, R., Fujioka, Y., Endo, Y., Ueki, M., et al. (2018). A Computer-Based Glucose Management System Reduces the Incidence of Forgotten Glucose Measurements: A Retrospective Observational Study. *Diabetes Ther.* 9, 1143–1147. doi: 10.1007/s13300-018-0427-z.

Park, J., Kim, J., Kim, S.-Y., Cheong, W. H., Jang, J., Park, Y.-G., et al. (2018). Soft, smart contact lenses with integrations of wireless circuits, glucose sensors, and displays. *Sci. Adv.* 4, eaap9841. doi: 10.1126/sciadv.aap9841.

Ribet, F., Stemme, G., and Roxhed, N. (2018). Real-time intradermal continuous glucose monitoring using a minimally invasive microneedle-based system. *Biomed. Microdevices* 20, 101. doi: 10.1007/s10544-018-0349-6.

Segman, Y. (Joseph) (2018). Device and Method for Noninvasive Glucose Assessment. *J. Diabetes Sci. Technol.* 12, 1159–1168. doi: 10.1177/1932296818763457.

Shaker, G., Smith, K., Omer, A. E., Liu, S., Csech, C., Wadhwa, U., et al. (2018). Non-Invasive Monitoring of Glucose Level Changes Utilizing a mm-Wave Radar System: *Int. J. Mob. Hum. Comput. Interact.* 10, 10–29. doi: 10.4018/IJMHCI.2018070102.

Shang, T., Zhang, J. Y., Thomas, A., Arnold, M. A., Vetter, B. N., Heinemann, L., et al. (2022). Products for Monitoring Glucose Levels in the Human Body With Noninvasive Optical, Noninvasive Fluid Sampling, or Minimally Invasive Technologies. *J. Diabetes Sci. Technol.* 16, 168–214. doi: 10.1177/19322968211007212.

Sim, J. Y., Ahn, C.-G., Jeong, E.-J., and Kim, B. K. (2018). In vivo Microscopic Photoacoustic Spectroscopy for Non-Invasive Glucose Monitoring Invulnerable to Skin Secretion Products. *Sci. Rep.* 8, 1059. doi: 10.1038/s41598-018-19340-y.

UBAND-Know Labs. Bio-RFID™. Transforming Non-Invasive Medical Diagnostics Available at: https://www.knowlabs.co.

Vega, K., Jiang, N., Liu, X., Kan, V., Barry, N., Maes, P., et al. (2017). The dermal abyss: interfacing with the skin by tattooing biosensors. in *Proceedings of the 2017 ACM International Symposium on Wearable Computers* (Maui Hawaii: ACM), 138–145. doi: 10.1145/3123021.3123039.

Villena Gonzales, W., Mobashsher, A., and Abbosh, A. (2019). The Progress of Glucose Monitoring—A Review of Invasive to Minimally and Non-Invasive Techniques, Devices and Sensors. *Sensors* 19, 800. doi: 10.3390/s19040800.
